# Mesenchymal stem cell-derived extracellular vesicles for dry eye disease: principles, progress, and challenges

**DOI:** 10.20517/evcna.2025.174

**Published:** 2026-06-24

**Authors:** Yuting Feng, Mingqi Zhang

**Affiliations:** ^1^School of Clinical Medicine, He University, Shenyang 110163, Liaoning, China.; ^2^Stem Cell Center of Precision Medicine Innovation Institute, He University, Shenyang 110001, Liaoning, China.; ^3^Liaoning Key Lab of Ophthalmic Stem Cells, He University, Shenyang 110001, Liaoning, China.; ^4^Shenyang He Eye Hospital, Shenyang 110034, Liaoning, China.

**Keywords:** Dry eye disease, mesenchymal stem cells, extracellular vesicles, inflammation, immunomodulation, exosomes

## Abstract

Dry eye disease (DED) is a multifactorial ocular surface disorder characterized by disruption of tear film and/or ocular surface homeostasis that represents a significant global public health concern. This review summarizes the pathogenesis of DED, with particular emphasis on the central role of ocular surface inflammation. It further comprehensively evaluates the therapeutic rationale, preclinical advances, and translational challenges associated with mesenchymal stem cell-derived extracellular vesicle (MSC-EV) therapy for DED. MSC-EVs exhibit numerous properties, including anti-inflammatory, immunomodulatory, reparative, and regenerative effects, and offer key advantages such as a cell-free nature, low immunogenicity, minimal tumorigenic risk, high stability, and suitability for topical administration. Through advanced strategies such as cargo engineering, hybrid design, biomaterial-assisted delivery, and genetic engineering, the therapeutic performance of MSC-EVs can be further optimized, enabling targeted delivery, improved retention and bioavailability, and precise immunomodulation. In addition, three-dimensional ocular surface and *ex vivo* models help overcome the limitations of traditional animal models in replicating human ocular physiology. Among available sources, umbilical cord-derived MSC-EVs represent one of the most promising candidates for clinical translation. Despite these advances, several challenges remain, including ocular-specific anatomical and physiological barriers, limited pharmacological characterization, lack of standardized large-scale production and storage protocols, incomplete toxicological and microbiological safety evaluation, and the absence of a unified regulatory framework. Overall, MSC-EVs represent a potentially transformative therapeutic strategy for the management of refractory DED. However, as most current evidence remains preclinical, further validation in human-relevant models and clinical studies is essential before definitive conclusions can be drawn.

## INTRODUCTION

Dry eye disease (DED) is a multifactorial disorder characterized by disruption of tear film and/or ocular surface homeostasis. Key contributing factors include tear film instability, hyperosmolarity, ocular surface inflammation, and neurosensory abnormalities^[1]^. Tear film instability and hyperosmolarity are considered the primary initiating factors that subsequently induce ocular symptoms and compensatory responses. If these pathological processes persist and progress through a cascade of events, chronic inflammation of the ocular surface may ensue, ultimately resulting in damage to epithelial tissues such as the cornea and conjunctiva^[2]^.

Common symptoms of DED include persistent ocular discomfort, pain, redness, excessive tearing, burning sensation, foreign body sensation, grittiness, photophobia, and decreased functional visual acuity. In advanced stages, the disease may progress to severe complications, such as corneal ulceration, perforation, or scarring, and may ultimately lead to vision impairment. With increasing prevalence across diverse age groups, DED has emerged as a major public health concern worldwide^[3,4]^.

Despite the widespread availability of conventional therapies such as artificial tear substitutes, topical anti-inflammatory agents, and immunosuppressive treatments - current management strategies remain largely focused on symptomatic relief or suppression of inflammation. However, these interventions fail to effectively restore ocular surface homeostasis or disrupt the underlying pathogenic mechanisms. Consequently, there is an increasing need for the development of more targeted therapeutic strategies.

In recent years, regenerative medicine has provided novel perspectives for the treatment of refractory ocular surface diseases. Among these emerging strategies, mesenchymal stem cells (MSCs) have been the most extensively studied in clinical trials. MSCs are multipotent stromal cells capable of self-renewal and differentiation in response to microenvironmental cues^[5,6]^. These cells are widely distributed across multiple tissues and organs, including bone marrow, adipose tissue, dental pulp, umbilical cord, amniotic fluid, placenta, and Wharton’s jelly^[7-13]^. In addition to participating in direct cell-cell interactions, MSCs release soluble immunomodulatory molecules and extracellular vesicles (EVs) via paracrine signaling to communicate with target cells. Through these mechanisms, MSCs exert multiple therapeutic effects, such as regulation of inflammation, angiogenesis, immune modulation, and tissue repair and regeneration^[5,6,10,14-19]^. Owing to their multi-mechanistic synergy, MSCs and MSC-derived EVs (MSC-EVs) hold substantial promise for the treatment of DED. However, MSC transplantation has several drawbacks: low survival rates, poor storage stability, scalability issues, tumorigenic and immunogenic risks, and ethical concerns. These limitations have shifted attention to MSC-EVs as a promising cell-free alternative^[5,20,21]^. This review summarizes the pathological mechanisms underlying DED, with an emphasis on the central role of inflammation, and outlines current advances in MSC-EV-based therapy for DED. It further discusses key challenges and future directions for clinical translation, particularly the ocular-specific anatomical and physiological barriers. By integrating the inflammatory cascade of DED with the multifaceted actions of MSC-EVs, this work aims to provide a conceptual framework for the development of next-generation therapeutic strategies. Based on the available literature and clinical studies, it is proposed that umbilical cord-derived MSC-EVs (UCMSC-EVs) may represent one of the most promising candidates for clinical translation in the treatment of DED.

## OVERVIEW OF DED

DED is a highly prevalent ocular disorder with substantial global distribution and notable demographic disparities. In the United States, the prevalence of clinically diagnosed DED among adults aged 18 years and older varies widely across study cohorts, ranging from 2.15% to 16.59%. The overall prevalence is markedly higher in females and rises progressively with age, although a considerable disease burden has also been reported in young adults aged 18-34 years^[22]^. After multivariate statistical adjustment, no significant differences have been observed with respect to race, educational level, or geographic region within the United States^[22]^. In Asian populations, the pooled prevalence of DED has been estimated at 20.1%, with a clear sex difference (21.7% in females *vs*. 16.4% in males). In China, the overall prevalence reaches 20.9% (95% credible interval: 13.5-30.9), similarly demonstrating age- and sex-related increasing trends^[23]^.

DED can be classified into aqueous-deficient dry eye (ADDE), evaporative dry eye (EDE), and mixed-type dry eye, the latter being the most common in clinical practice. ADDE is further subdivided into Sjögren’s syndrome-related (primary or secondary) and non-Sjögren’s syndrome-related forms. Secondary Sjögren’s syndrome may arise from systemic autoimmune diseases, including rheumatoid arthritis, systemic lupus erythematosus, and systemic sclerosis^[1,24]^. Additional causes of ADDE include aging, lacrimal gland dysfunction, inflammation, lacrimal duct obstruction due to scarring, ocular surgery (such as refractive, cataract, glaucoma, and retinal surgery, as well as intravitreal injections), and the use of certain medications (e.g., anticholinergic drugs, hormone replacement therapy, diuretics, isotretinoin, and topical eye drops)^[3,24-26]^.

**Figure 1:**
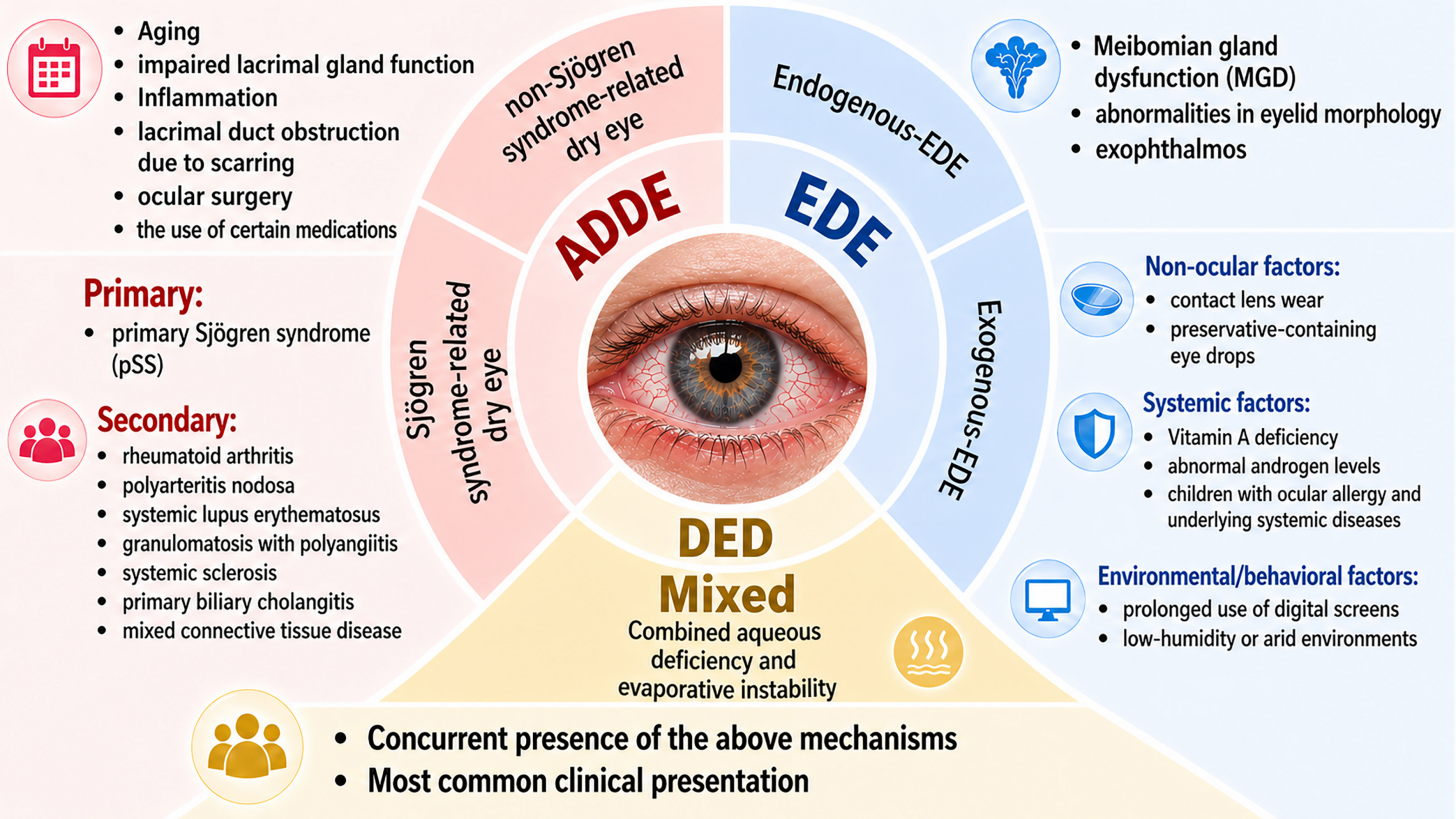
Classification and common etiologies of dry eye disease. ADDE: Aqueous-deficient dry eye; EDE: evaporative dry eye; DED: dry eye disease.

EDE arises from both endogenous (ocular) and exogenous (non-ocular) factors. The primary driver is meibomian gland dysfunction, which reduces tear film lipids and accelerates evaporation^[24]^. Additional endogenous factors include eyelid abnormalities that impair normal eyelid closure, reduced blink frequency (common in the elderly)^[1,24]^, and exophthalmos associated with thyroid eye disease^[27]^. Exogenous factors disrupt the balance in the ocular surface microenvironment. Common exogenous factors include contact lens wear, which can damage goblet cells and reduce mucin by mechanical friction^[28]^, and long-term use of preservative-containing eye drops. Systemic conditions also play a role, such as vitamin A deficiency (impairing mucin production)^[29,30]^, and androgen imbalance, which affects lacrimal and meibomian gland function and partly explains the higher prevalence in females^[24,31]^. In pediatric populations, risk factors include ocular allergy and systemic diseases^[32]^. Environmental and behavioral factors include prolonged screen time, low-humidity indoor environments (e.g., air-conditioned offices), and arid workplaces^[24,33-37]^. A summary of DED types and their typical causes is presented in Figure 1^[1,3,24]^.

## PATHOGENESIS OF DED

**Figure 2:**
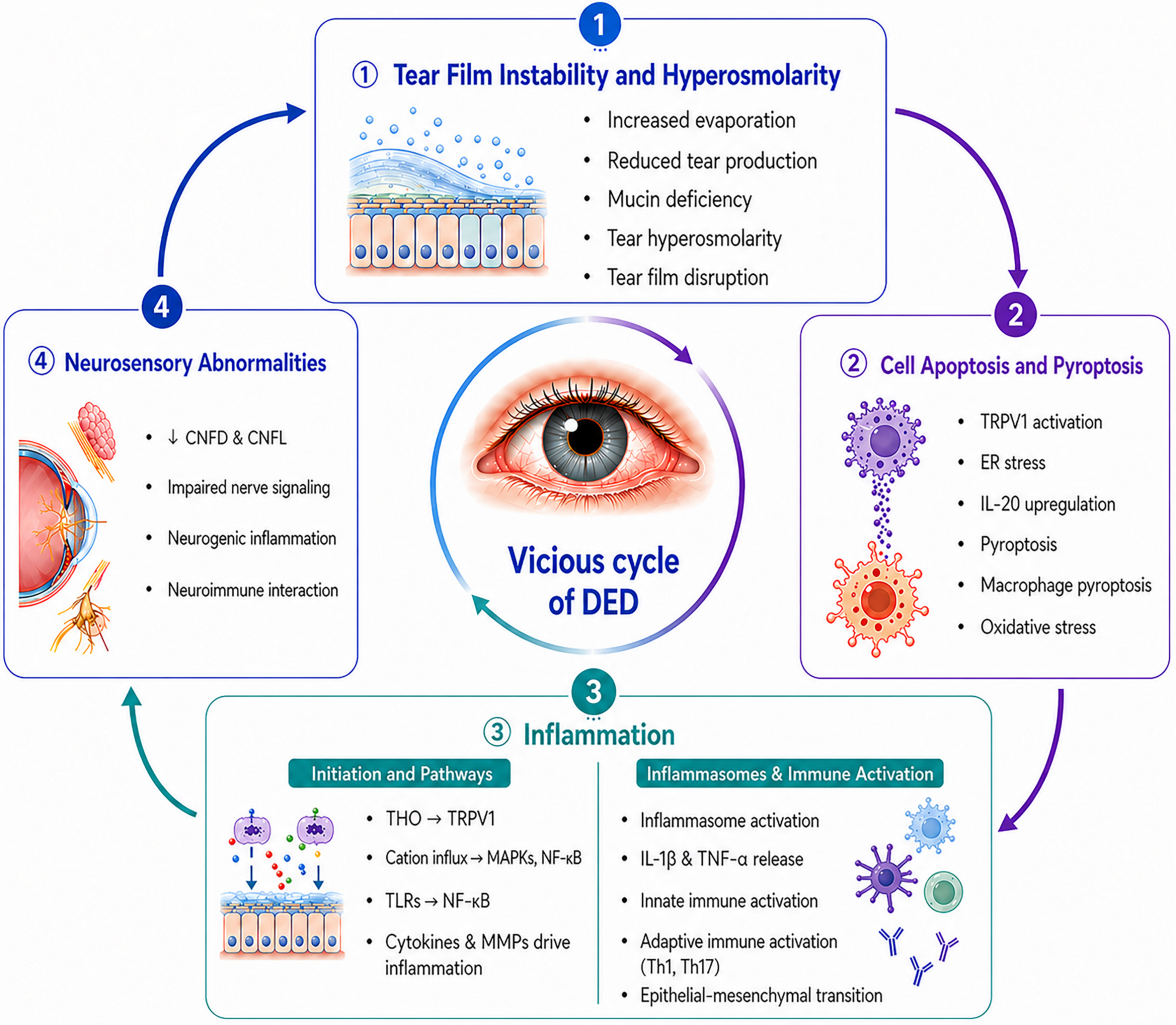
The vicious cycle in the pathogenesis of dry eye disease. CNFD: Corneal nerve fiber density; CNFL: corneal nerve fiber length; DED: dry eye disease; ER: endoplasmic reticulum; IL-20: interleukin 20; IL-1β: interleukin-1beta; MAPKs: mitogen-activated protein kinases; MMPs: matrix metalloproteinases; NF-κB: nuclear factor kappa-light-chain-enhancer of activated B cells; THO: tear hyperosmolarity; TLRs: Toll-like receptors; TNF-α: tumor necrosis factor alpha; TRPV1: transient receptor potential vanilloid 1.

The pathogenesis of DED involves a multiphase cascade reaction. It typically begins with tear film instability, which leads to increased tear hyperosmolarity (THO). This hyperosmolar environment subsequently triggers ocular surface inflammation, epithelial apoptosis, and pyroptosis, ultimately resulting in tissue damage. And here’s the catch: the damaged ocular surface makes tear film instability even worse, creating a self-perpetuating vicious cycle that drives disease progression and chronicity [Figure 2]. As the disease advances, tear secretion progressively declines, resulting in the characteristic symptoms and signs of DED^[24,25]^.

### Tear film instability and THO

Wolff first proposed the classic three-layer tear film model back in 1946. It consisted of an outer lipid layer that prevents tear evaporation, a middle aqueous layer responsible for ocular lubrication and nutritional supply, and an inner mucin layer that enables firm adhesion to the hydrophobic corneal epithelium^[4,38]^. Newer models favor a simpler bilayer structure: a lipid layer on top of a combined mucin-aqueous layer, with the mucin-aqueous part making up most of the tear film^[39,40]^. The lacrimal glands secrete most of the tear fluid, while meibomian glands produce the outer lipid layer, collectively maintaining ocular surface health.

Both ADDE and EDE can disrupt tear film stability. Tears are primarily saline solutions containing electrolytes, proteins, and other solutes. Alterations in tear composition or secretion volume increase tear osmotic pressure, thereby inducing THO, a core trigger of DED. Notably, THO correlates strongly with tear film instability^[41]^. During periods of tear film instability, rapidly fluctuating tear osmolarity may reach peak levels, activating corneal nerve endings and ocular surface cells and triggering discomfort and pro-inflammatory signaling pathways^[41-43]^. Normal tear osmolarity is around 302 ± 9.7 mOsm/L. When corneal epithelial cells are exposed to hyperosmotic environments, signaling pathways that mediate inflammatory responses are activated^[44]^. The response threshold of corneal sensory neurons to increased tear osmolarity is approximately 450 mOsm/kg and above^[41]^. Elevated osmolarity triggers ion channel-mediated pathways in corneal epithelial cells and eye-infiltrated immune cells, stimulating transcriptional factors that upregulate the expression of inflammatory cytokines, ultimately eliciting robust ocular surface inflammation. Collectively, tear film instability and THO are regarded as the key initiating factors in the development and progression of DED^[45-47]^.

### Cell apoptosis and pyroptosis

Exposure to a hyperosmotic environment creates a persistent osmotic imbalance in which the tear film’s osmolarity exceeds that of intracellular fluid, leading to epithelial cell shrinkage and increased intracellular solute concentration. In DED, hyperosmotic and desiccating stresses promote apoptosis and pyroptosis of ocular surface epithelial cells^[24]^. The mechanisms include the following.

(1) Activation of the transient receptor potential vanilloid 1 (TRPV1) channel and downstream pathways Hyperosmolar stress activates the TRPV1 channel in corneal epithelial cells, initiating the transcription factor nuclear factor kappa-light-chain-enhancer of activated B cells (NF-κB) signaling cascade^[19,46,48,49]^. Additionally, the mitogen-activated protein kinase (MAPK) pathways^[46,48,49]^, including c-Jun N-terminal kinases (JNK) and p38, are also activated^[49]^. The NF-κB, JNK, and p38 are critically involved in cellular stress responses and the induction of apoptosis^[19,50,51]^.

(2) Endoplasmic reticulum (ER) stress and unfolded protein response (UPR) Hyperosmolarity induces ER dysfunction, leading to apoptosis in corneal and conjunctival epithelial cells. In Sjögren’s syndrome-related DED patients and hypertonic stress models, genes associated with the UPR and the transcription factor DDIT3 (DNA-damage-inducible transcript 3), which promotes apoptosis, are significantly upregulated. THO-induced ER stress enhances UPR activation, which in turn promotes DDIT3 expression, ultimately triggering apoptosis to eliminate stressed epithelial cells of the cornea and conjunctiva^[42]^.

(3) Upregulation of interleukin (IL)-20 via nuclear factor of activated T-cells 5 (NFAT5) activation Through activation of the osmosensitive transcription factor NFAT5, also called TonEBP, hyperosmolarity upregulates IL-20 expression in tear samples from DED patients and in the tears and corneal tissues from DED animal models^[52,53]^. IL-20 may act as a pro-inflammatory mediator that amplifies immune responses by promoting macrophage activation and inducing cell death, thereby exacerbating DED progression^[52]^.

(4) Inflammasome-mediated pyroptosis Hyperosmolarity activates multiple inflammasomes, including NOD-like receptor protein 3 (NLRP3), NLRP12, and NOD-like receptor CARD domain-containing 4 (NLRC4), leading to pyroptosis^[54-56]^, a form of inflammatory cell death characterized by gasdermin D (GSDMD)-mediated cell lysis. Increased pyroptosis via the NLRP3/apoptosis-associated speck-like protein containing a CARD (ASC)/Caspase-1/GSDMD axis has been detected in both hyperosmotic stress-stimulated human corneal epithelial cells and tear samples from DED patients^[55]^. In DED, NLRP12 and NLRC4 inflammasomes regulate each other and facilitate pyroptosis under the control of CASP1-triggered GSDMD cleavage^[56]^. Additionally, interferon gamma (IFN-γ) further promotes pyroptosis via Janus kinase 2 (JAK2)/signal transducer and activator of transcription (STAT) 1-dependent NLRP3 activation^[57]^.

(5) Lactate-reactive oxygen species (ROS) axis and macrophage pyroptosis Hyperosmotic stress reprograms the metabolism of corneal epithelial cells, leading to lactate accumulation. This accumulation subsequently promotes the generation of ROS in macrophages, activates the NLRP3/caspase-1/IL-1β pathway, and ultimately induces macrophage pyroptosis, and the release of the pro-inflammatory cytokine IL-1β, thereby amplifying ocular surface inflammation^[58]^.

(6) Oxidative stress and antioxidant imbalance Hyperosmolarity also induces oxidative stress by disrupting redox homeostasis, which is characterized by elevated intracellular ROS levels and suppression of key antioxidant enzymes such as superoxide dismutase (SOD) and catalase (CAT), ultimately resulting in apoptosis and pyroptosis. Concurrently, the level of malondialdehyde (MDA), an oxidative stress biomarker, increases significantly. As an endogenous product of lipid peroxidation, MDA can react with deoxyribonucleotides to produce a variety of adducts and damage DNA^[57,59-61]^. *In vitro*, hyperosmotic conditions induced by 50 or 100 mM sodium chloride cause morphological damage, reduced cell viability, increased apoptosis, and mitochondrial membrane potential alterations in human corneal epithelial cells^[59,61]^. Decreased SOD and CAT activity impairs antioxidant defense, exacerbating ROS-mediated damage^[59]^. ROS accumulation in ocular tissues not only directly damages nucleic acids, proteins, and lipids but also acts as an upstream mediator of inflammatory responses and THO, ultimately disrupting cellular homeostasis^[60,62]^.

Collectively, THO serves as a potent initiator of corneal and conjunctival epithelial apoptosis and pyroptosis, disrupting goblet cell function and corneal barrier integrity^[41,43,51]^. Moreover, prolonged air exposure can directly damage the corneal epithelium, which serves as a critical element in both the onset and progression of DED. Associated symptoms may include ocular dryness, corneal epithelial defects, chronic inflammation, visual impairment, and even blindness^[63]^.

### Inflammation

#### Initiation and signaling pathways

THO initiates inflammatory signaling cascades by activating ion channel-mediated pathways in both corneal epithelial cells and infiltrating immune cells within the ocular surface. This results in activation of transcription factors that upregulate genes encoding inflammatory cytokines, ultimately leading to robust inflammatory responses in DED patients. Persistent uncontrolled immune activation disrupts the structure and function of ion channel proteins, thereby reinforcing THO and worsening symptoms^[45]^. Corneal epithelial cells and sensory neurons respond to hyperosmolarity at thresholds of approximately 600-700 mOsm/kg. Neurons mediate nociceptive signaling, whereas epithelial cells activate inflammatory signaling^[41]^.

**Figure 3:**
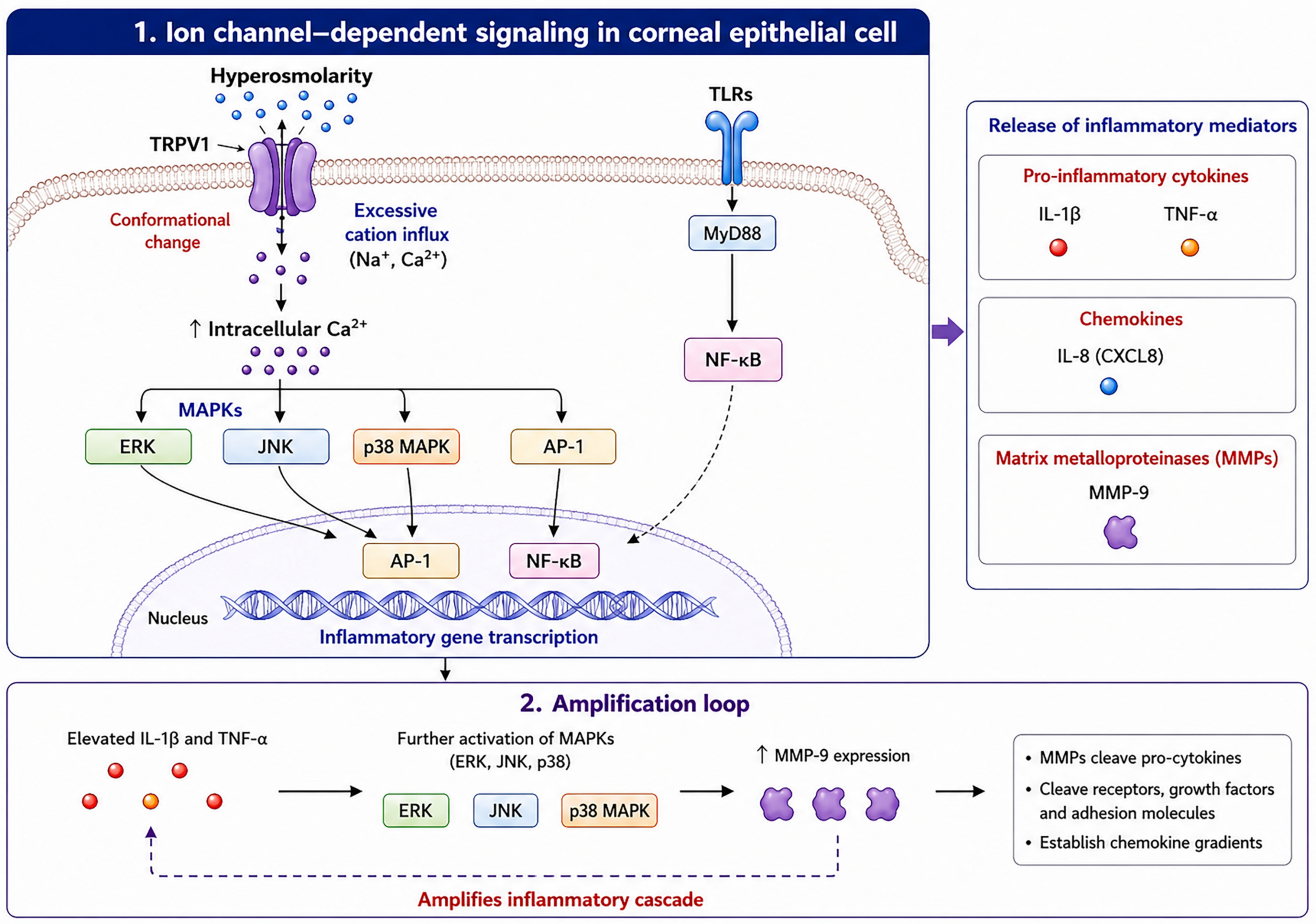
Initiation and Signaling Pathways of Inflammation in DED. AP-1: Activator protein 1; CXCL8: C-X-C motif chemokine ligand 8; ERK: extracellular signal-regulated kinase; IL-1β: interleukin-1 beta; IL-8: interleukin-8; JNK: c-Jun N-terminal kinase; MAPKs: mitogen-activated protein kinases; MMP-9: matrix metalloproteinase 9; MyD88: myeloid differentiation primary response 88; NF-κB: nuclear factor kappa-light-chain-enhancer of activated B cells; p38 MAPK: p38 mitogen-activated protein kinase; TLRs: Toll-like receptors; TNF-α: tumor necrosis factor alpha; TRPV1: transient receptor potential vanilloid 1.

THO induces conformational changes in TRPV1 channels, allowing excessive cation influx and disrupting cellular homeostasis^[45,64]^. Increased intracellular calcium, often partly via TRPV1, subsequently activates transcription factors such as MAPKs, activator protein 1 (AP-1), and NF-κB. These events trigger the secretion of pro-inflammatory cytokines, chemokines, and matrix metalloproteinases (MMPs), escalating inflammatory responses in a cascade-like manner^[1,19,48,49,51,65-68]^. MAPKs constitute a family of highly conserved signaling pathways consisting of three major members: p38 MAPK, extracellular signal-regulated kinase (ERK), and JNK. By activating MAPK pathways, hyperosmolarity induces a pro-inflammatory response in both human limbal epithelial cells and corneal epithelial cells via IL-1β, tumor necrosis factor-α (TNF-α), MMP-9, and the C-X-C chemokine IL-8^[19,51,69-71]^. Elevated IL-1β and TNF-α further drive MAPK activation and promote MMP-9 expression^[71]^. In DED, inflammatory cascades are promoted by MMPs, which act through cleavage of pro-cytokines and additional extracellular proteins (e.g., receptors, growth factors, adhesion molecules) and through the establishment of chemokine gradients^[72]^. The levels of MMP-9, one of the most important MMPs on the ocular surface^[71]^, correlate positively with tear osmolarity, for which it serves as a potential biomarker^[65]^. Additionally, Toll-like receptors (TLRs) on epithelial cells activate NF-κB via the myeloid differentiation primary response protein 88 (MyD88)-dependent pathway, further amplifying inflammatory gene expression^[73,74]^. Figure 3 summarizes the initiation and signaling pathways of inflammation in DED.

#### Innate and adaptive immune activation and the inflammasome

Hyperosmotic and desiccating stress induces ROS in human corneal epithelial cells, promoting IL-1β production via the downstream NLRP3/caspase-1/IL-1β axis^[54]^. Additionally, TLR4 stimulation by desiccating stress could activate the NLRP12 and NLRC4 inflammasomes via caspase-8 signaling^[56]^. Inflammasomes serve as critical elements of innate immunity, facilitating the maturation and secretion of IL-1β and IL-33^[1,51,55,56,58,75-77]^. IL-1β bridges innate and adaptive immunity, serving as a pro-inflammatory hallmark of DED onset and progression^[75]^. Similarly, IL-33, an important member of the IL-1 family, serves as a pro-inflammatory cytokine that mediates ocular surface alterations in DED^[56]^.

IL-1β and TNF-α stimulate multiple kinases and transcription factors within dendritic cells (DCs) infiltrating the eye, thereby driving DC-mediated naïve T cell differentiation into Th1 and Th17 subsets^[68]^. A cascade of kinase activity leads to phosphorylation and activation of the transcriptional regulators c-Jun and activating transcription factor 2 (ATF2). Following activation, these two regulators migrate to the nucleus and upregulate the production of pro-Th1 (IL-12) and pro-Th17 (IL-1β, IL-6, IL-23) cytokines in DCs. IL-12 released by DCs activates the T-box protein expressed in T cells (T-bet) as well as STAT1 in naïve CD4^+^ T lymphocytes, consequently steering them toward an IFN-γ-producing Th1 phenotype. Likewise, IL-1β, IL-6, and IL-23 released by DCs activate STAT3 and RAR-related orphan receptor gamma T (RORγT) in naïve CD4^+^ T cells, fostering the growth and maturation of inflammatory CD4^+^ Th17 cells capable of producing IL-17 and IL-22^[19,68,78]^.

Th1-derived IFN-γ together with Th17-derived IL-17 and IL-22 further stimulates macrophages and neutrophils on the ocular surface in DED patients. This induces M1 macrophage polarization and release of pro-inflammatory cytokines, chemokines, MMPs, adhesion molecules, and pro-lymphangiogenic molecules, followed by recruitment of monocytes and neutrophils to the inflammatory site^[72,79]^. Th1 C-X-C motif chemokine ligands CXCL9, CXCL10, and CXCL11 and Th17 C-C motif chemokine ligand CCL20, released by M1 macrophages and neutrophils, recruit and anchor circulating Th1 and Th17 cells to the ocular inflammation sites. This process subsequently results in substantial infiltration of CD4^+^ Th1/Th17 cells into the inflamed ocular surface in DED patients^[79-81]^. Under physiological conditions, regulatory T cells (Tregs) help maintain immune homeostasis and promote peripheral tolerance^[78,82]^; however, an imbalanced Th17/Treg ratio disrupts this equilibrium and contributes to disease progression^[73]^. The resulting Th1- and Th17-mediated inflammation drives tissue damage through distinct but complementary mechanisms. Th1-type inflammation promotes apoptosis of ocular epithelial cells, squamous metaplasia, and goblet cell loss, whereas Th17 cytokines damage lacrimal and meibomian glands and reduce mucin production. In parallel, activation of MMPs contributes to disruption of the corneal epithelial cell barrier^[46,72,80,83]^. The ocular surface pathology in DED promoted by M1 macrophages and neutrophils is characterized by diminished mucin secretion and the combined loss of corneal epithelial cells, conjunctival epithelial cells, and goblet cells through apoptosis^[79]^.

Repeated desiccating stress also induces epithelial-mesenchymal transition, generating fibroblast-like epithelial cells enriched in IL-2/STAT5 signaling, IL-6/JAK/STAT3 signaling, and the inflammatory response pathway, with increased levels of multiple chemokines, including CXCL1, CXCL10, CCL2, and CCL19. These findings suggest that fibroblast-like epithelial cells primarily recruit immune cells and promote acute inflammation in DED. The crosstalk between these cells and macrophages/CD4^+^ T lymphocytes constitutes a critical basis for the subsequent inflammatory cascades in DED^[84]^.

In summary, the pathogenesis of DED involves the dual activation of innate and adaptive immunity^[85]^. Under THO and desiccating stress, the damaged corneal epithelium releases inflammatory cytokines that promote the maturation of resident antigen-presenting cells. These cells subsequently capture antigens and migrate to draining lymph nodes, where they activate naïve T cells that then home to the ocular surface^[46,81,85,86]^. Infiltrating T cells cause further tissue injury and reactivate innate inflammation, creating a self-sustaining cycle of “inflammation → damage → re-inflammation” that drives DED onset and persistence^[85,87]^.

### Neurosensory abnormalities

The lacrimal functional unit, comprising the ocular surface, lacrimal glands, and neural connections, is essential for ocular surface homeostasis^[24,51]^. The cornea is densely innervated, predominantly by sensory nerve fibers from the ophthalmic branch of the trigeminal ganglion, which mediate essential physiological functions such as thermosensation, mechanosensation, nociception, the blink reflex, tear secretion, and wound healing^[88,89]^. Corneal nerves are therefore crucial for maintaining ocular surface health and homeostasis^[90]^. Subliminal stimulation of corneal nerves generates afferent signals via the trigeminal nerve, which are integrated within the central nervous system to produce efferent secretomotor impulses that stimulate tear secretion^[87]^.

Corneal nerve abnormalities are prevalent in DED^[89,91]^. THO alone is sufficient to induce a reduction in corneal intraepithelial nerves and terminals, and sensitize the ocular surface to hypertonicity^[1,46]^. In earlier stages of the inflammatory cycle, ocular surface damage leads to a compensatory reflex stimulation of the lacrimal gland^[89]^. In DED models, excessive reflex stimulation of the lacrimal gland can produce a neurogenic pro-inflammatory cytokine response that includes: (1) release of inflammatory markers into tears; (2) significant upregulation of IL-6 and IL-1β mRNA in the trigeminal ganglion; and (3) elevated inflammatory cytokines (IL-6, TNF-α, IL-1β, CCL2) in the trigeminal brainstem sensory complex. As disease progresses, inflammatory dendritic cell infiltration increases, accompanied by a decrease in corneal nerve fiber density and length^[91-93]^. Increased spontaneous activity in ciliary nerves reported in DED mice indicates that persistent dryness induces corneal inflammation, affecting nociceptor activity and leading to peripheral sensitization and corneal hypersensitivity. Additionally, the corneal nerves exhibited morphological changes, notably fewer terminals in the apical region of the corneal epithelium^[89]^. Compared to healthy subjects, DED patients exhibit lower total, main, and branch nerve numbers and lower nerve density in the corneal subbasal nerves and these changes are more pronounced in aqueous-deficient DED^[94]^. Impaired corneal nerve reflexes further reduce tear secretion, exacerbating inflammation. In addition, inflammatory stimuli may cause an increase or dysregulation in the sensitivity of nerve terminals, resulting in symptoms such as overt pain and a burning sensation in some patients.

Beyond indirect neuroimmune regulation, recent evidence suggests contact-mediated mechanisms, whereby direct neuronal interactions modulate the kinetics and morphology of conventional DCs in the cornea of DED mice, indicating a direct neural modulation of immune responses^[95]^.

Neurosensory abnormalities are therefore both drivers and consequences of the inflammatory vicious cycle in DED, contributing to hypersensitivity, pain, dysfunction of the tear reflex arc, and amplified local immune responses. Understanding the bidirectional neuro-immune feedback loop is essential for the development of comprehensive therapeutic strategies.

## MSC-DERIVED EVs FOR THE TREATMENT OF DED

Current clinical management of DED provides only limited relief from ocular surface discomfort and inflammatory injury^[19,26,62,96-98]^. Most existing therapies primarily focus on symptom control rather than addressing the underlying pathogenic mechanisms. So, the advancement of regenerative approaches that target the core pathophysiology of DED has become an urgent priority. Recently, MSC-EVs have attracted increasing attention for their powerful abilities to promote tissue repair, regulate immune responses, and resolve inflammation across various inflammatory and autoimmune conditions^[12,99,100]^. MSC-EVs represent a promising novel therapeutic strategy for DED due to their robust immunomodulatory capacity, low immunogenicity, and the ability to simultaneously regulate both innate and adaptive immune responses^[6,101]^.

### Treatment of DED with MSC-EVs: underlying principles

EVs are membrane-bound nanoparticles released by both eukaryotic and prokaryotic cells. The lipid bilayer membrane of these vesicles is characterized by high levels of cholesterol, sphingomyelin, ceramide, and lipid raft proteins, and encapsulates a wide range of bioactive cargos, such as proteins, lipids, and nucleic acids^[99,102-106]^. Based on their origin and size, EVs are commonly classified into three major subtypes^[102,107]^: (1) exosomes (30-200 nm in diameter) are endosome-derived vesicles generated through inward budding of multivesicular bodies and their fusion with the plasma membrane; (2) microvesicles (100-1,000 nm in diameter) originate from outward budding and scission of the plasma membrane; (3) apoptotic bodies (> 1,000 nm in diameter) are shed from cells that are undergoing programmed cell death. The size distributions of these EV subtypes often overlap partially. Once released, exosomes are internalized by recipient cells in the local microenvironment or transported systemically via the circulatory system^[102]^. EVs facilitate intercellular communication by transferring bioactive cargo from donor to recipient cells via mechanisms such as direct membrane fusion, receptor-ligand interactions, endocytosis, and phagocytosis^[99,102,104,108-110]^. After internalization, the encapsulated bioactive molecules are released into the cytoplasm of recipient cells^[102]^.

MSC-EVs carry a rich array of bioactive substances, including enzymes, cytokines, chemokines, growth factors, and ribonucleic acids (RNAs) such as messenger RNAs (mRNAs) and regulatory microRNAs (miRNAs)^[107,111-118]^ that allow them to participate in intercellular signaling, homeostasis maintenance, angiogenesis, immune regulation, and inflammatory control^[119-122]^. MSC-EVs have emerged as an innovative and highly promising treatment strategy for ocular surface diseases due to their cell-free nature, low tumorigenic risk, superior immune tolerance, and potent bio-regulatory capabilities^[101]^.

#### The anti-inflammatory and immunomodulatory effects of MSC-EVs in the treatment of DED

MSC-EVs exert potent anti-inflammatory and immunoregulatory effects by transferring bioactive molecules, including regulatory miRNAs and immunomodulatory proteins, into infiltrating immune cells at the ocular surface^[102,123-125]^. miRNAs are small non-coding RNA molecules, typically 20-22 nucleotides in length, that modulate gene expression by influencing the stability of mRNAs and interfering with protein biosynthesis, consequently altering the phenotype and function of immune cells^[126]^.

Suppression of inflammation via targeting of core inflammatory pathways A key target of MSC-EVs in DED therapy is the NF-κB pathway. This pathway is a master regulator of inflammation, immune response, and cell survival. The canonical NF-κB signaling cascade involves key components such as TLR4, IL-1 receptor-associated kinase 1 (IRAK1), TNF receptor-associated factor 6 (TRAF6), transforming growth factor beta 1 (TGF-β) activated kinase 1/MAP3K7 binding protein 2 (TAB2), and NF-κB inhibitor epsilon (NFKBIE), among others^[127]^.

Several studies show that human UCMSC-EVs are enriched with miRNAs such as miR-125b, let-7b, and miR-6873, that hit multiple upstream parts of the NF-κB pathway. In a DED mouse model, these miRNAs target TLR4, IRAK1, TAB2, and TRAF6, reversing the pathway’s abnormal activation. This multi-pronged inhibition sharply lowers pro-inflammatory mediators (like TNF-α, IL-4, IL-8, IL-10, and IL-17) while boosting protective cytokines such as IL-13 in the cornea and conjunctiva. Tear cytokine profiling further confirmed a broad drop in inflammatory cytokines, including IL-2, IL-6, IL-7, IL-11, IL-12p70, IL-15, IL-17, IL-1Ra, IL-10, TNF-α, IFN-γ, IL-5, and the chemokines CCL2, CXCL1, and IL-8. Conversely, the tissue inhibitors of MMPs, TIMP-1 and TIMP-2, were markedly elevated. Collectively, these findings indicate that topical administration of human UCMSC-EVs alleviates DED symptoms, suppresses ocular inflammation, and restores corneal epithelial homeostasis through multi-targeted modulation of the IRAK1/TAB2/NF-κB pathway^[127]^. Likewise, bone marrow mesenchymal stem cell-derived exosomes (BMSC-Exos) deliver miR-21-5p to inhibit the TLR4/MyD88/NF-κB pathway. BMSC-Exo treatment significantly lowers tear and peripheral blood IL-17 and IL-22 concentrations while raising those of IL-4, IL-10, and TGF-β1, ultimately alleviating symptoms in both DED patients and mouse models^[73]^.

In Sjögren’s syndrome-associated DED models, lacrimal gland injection of MSC-EVs significantly downregulated the mRNA levels of pro-inflammatory mediators (TNF-α, IL-1β, and IFN-γ) in both the ocular surface and lacrimal glands while mitigating T-cell infiltration within the lacrimal glands^[106]^. Mechanistically, MSC-EVs inhibited the downstream TLR4 and T cell receptor signaling pathways in activated mouse splenocytes, thereby inhibiting the nuclear translocation of nuclear factor of activated T-cells 1 (NFAT1) and NF-κB. In TLR4-stimulated splenocytes, MSC-EVs also inhibited the nuclear translocation of NF-κB and p38. Notably, let-7b-5p encapsulated in the MSC-EVs was identified as a negative regulator of the TLR4/NF-κB pathway^[106]^. Levels of the downstream effector MMP-9 can increase through MAPK signaling activation by ocular epithelial and immune-cell-derived pro-inflammatory cytokines such as IL-6^[128,129]^. Clinically, topical ophthalmic administration of human Wharton’s jelly-derived MSC exosomes (WJMSC-Exos) reduced the gene expression of pro-inflammatory cytokines (such as IL-6 and IL-22) as well as MMP-9 in patients with Sjögren’s syndrome-associated DED^[128]^. Tear MMP-9 levels dropped one month after treatment, suggesting suppression of extracellular matrix (ECM) degradation and inflammation.

Additionally, miR-223-3p delivered in mouse adipose-derived MSC exosomes (ADMSC-Exos) has demonstrated potent anti-inflammatory effects in DED models through targeting F-box and WD repeat domain-containing 7 (Fbxw7) and subsequent significant reduction of pro-inflammatory cytokines (IL-6, IL-17, TNF-α, and IL-1β) and chemokines (CCL2 and CXCL1)^[130]^. Based on existing evidence that deficiency of the ubiquitination of enhancer of zeste homolog 2 (EZH2) promotes TNF-α-triggered NF-κB activation in intestinal epithelial cells, it was hypothesized that miR-223-3p may block EZH2 degradation by targeting the suppression of Fbxw7 expression, consequently inhibiting the inflammatory response in a DED model. Moreover, miR-223 has been shown to directly suppress NLRP3 inflammasome activity by inhibiting NLRP3 translation, thereby suppressing the inflammatory response in DED^[76]^. Several studies further confirm that adipose-derived MSC-derived EVs (ADMSC-EVs) can inhibit the assembly of desiccation stress-induced NLRP3 inflammasomes, the activation of caspase-1, and the maturation of IL-1β^[131-133]^. By suppressing the NLRP3/IL-1β signaling axis, inflammatory cytokines (e.g., IL-1β, IL-6, IL-1α, IFN-γ, and TNF-α) were reduced while the anti-inflammatory cytokine IL-10 was elevated, effectively mitigating DED-related ocular surface damage.

Immunomodulatory effects of MSC-EVs in the treatment of DED MSC-EVs can suppress DC-mediated inflammation, thereby alleviating ocular surface immune responses in DED. MSC-EVs have a general inhibitory effect on DCs. They inhibit the antigen uptake capacity of immature DCs, alter their morphology, reduce their dendrite number, and suppress both the increase in DC population and their maturation in DED. Consequently, the cytokine production profile of DCs shifts from an inflammatory to an immunoregulatory phenotype. MSC-EVs downregulate pro-inflammatory cytokines such as IL-6 and IL-12p70 and upregulate the anti-inflammatory cytokine TGF-β, thereby inhibiting DC proliferation and migration^[14,134]^. Moreover, miR-21-5p enriched in MSC-EVs may contribute to this inhibitory effect on DC function by downregulating CCR7 expression and impairing the chemotactic response to CCL21^[14]^. Collectively, these effects reduce Th17 cell activation, a key driver of ocular surface inflammation in DED.

Macrophages are critical innate immune cells with high plasticity and play dual roles in inflammation and tissue repair. Their specific functions are determined by their polarization status, which is shaped by signals from the local microenvironment. Conventionally, macrophages are divided into a pro-inflammatory M1 (classically activated) and an anti-inflammatory M2 (alternatively activated) phenotype^[135-137]^. MSC-EVs can suppress the activation of pro-inflammatory M1 macrophages while driving their polarization towards the anti-inflammatory M2 phenotype^[21,135]^. Human UCMSC small EVs (UCMSC-sEVs) mitigate autoimmune-mediated dacryoadenitis, possibly through delivering encapsulated miR-100-5p. This miRNA induces M2-polarized macrophages and enhances Treg populations *in vivo* and *in vitro*, thereby reducing inflammation and facilitating tissue repair. Following human UCMSC-sEV treatment in rabbit lacrimal glands, the mRNA levels of M1-associated genes - nitric oxide synthase 2 (*NOS2*), interferon-regulatory factor 5 (*IRF5*), *TNF-α*, *IL-1β*, and *IL-6* - were markedly reduced, whereas those of M2-related genes such as arginase-1 (*Arg1*), mannose receptor (*CD206*), Krueppel-like factor 4 (*KLF4*), *IL-10*, and *TGF-β* were significantly upregulated^[135]^. M1 macrophages produce high levels of inflammatory mediators, including TNF-α, IL-1β, IL-6, IL-12, and IL-23. These cytokines collectively serve as master regulators of inflammatory responses, while also recruiting and activating additional immune cells and ultimately contributing to tissue damage. In contrast, M2 macrophages predominantly produce anti-inflammatory cytokines such as IL-10 and TGF-β, which suppress inflammation and promote tissue repair^[136]^. In patients with graft-versus-host disease-associated DED refractory to currently available drugs or surgeries, eye drops containing exosomes from mesenchymal stromal cells (MSC-Exo) suppressed the infiltration and activation of ocular surface macrophages. MSC-Exo effectively regulated the phenotypic switching of macrophages, suppressed the expression of pro-inflammatory genes (e.g., IL-6, IL-1β, IL-17A, CD86), reestablished immune balance on the ocular surface, and consequently alleviated inflammatory damage. Mechanistically, MSC-Exo containing miR-204 directly engages interleukin-6 receptor (IL-6R), mediating the downregulation of IL-6/IL-6R/STAT3 signaling, which then induces the phenotypic switching of pro-inflammatory M1 macrophages to anti-inflammatory M2 macrophages on the ocular surface and thus alleviates dry eye symptoms^[21]^. The uptake of exosomes derived from periodontal ligament mesenchymal stem cell (PDLSC-Exos) by inflammatory M1 macrophages promotes their polarization toward the immunosuppressive M2 phenotype, characterized by elevated expression of IL-10 and Arg1. This polarization shift partially contributes to the protective effect of PDLSC-Exos on conjunctival goblet cells against M1 macrophage-mediated inflammation^[137]^.

MSC-EVs can reduce neutrophil infiltration in the corneas of rats with DED. Specifically, umbilical cord MSC-derived exosomes (UCMSC-Exos) disrupt neutrophil recruitment through multiple mechanisms, including suppression of chemotaxis, reduction of neutrophil extracellular trap (NET) formation, and modulation of apoptosis^[129]^.

MSC-EVs can significantly suppress CD4^+^ T cell proliferation and regulate their differentiation. Tregs, which characteristically express the forkhead transcription factor Foxp3, possess immunosuppressive functions. By increasing Treg populations while inhibiting Th17 cells, the Th17/Treg balance is effectively restored, ultimately leading to a marked immunosuppressive effect^[135]^. For instance, subconjunctival injection of human UCMSC-sEVs promoted an M1-to-M2 macrophage phenotypic shift in a rabbit model of dry eye, thereby suppressing the proliferation of CD4^+^ T cells and increasing the proportion of CD4^+^ Foxp3+ Tregs. MiR-100-5p has been identified as being at least partially responsible for the T cell suppression and Treg expansion mediated by macrophages incubated with human UCMSC-sEVs^[135]^. Further supporting their role in T cell modulation, human UCMSC-EVs were shown to inhibit the infiltration of CD4^+^ T cells into the conjunctiva of DED mice^[127]^. Similarly, BMSC-Exos alleviate DED progression through restoring the Th17/Treg balance by inhibition of the TLR4/MyD88/NF-κB pathway in CD4^+^ T cells. BMSC-Exo treatment significantly reduced levels of Th17 marker cytokines (IL-17 and IL-22) while increasing those of Treg-related cytokines (IL-4, IL-10, TGF-β1) in both tear fluid and peripheral blood of DED mice. Concurrently, the population of Th17-positive cells diminished and that of Treg-positive cells expanded, thereby restoring the Th17/Treg ratio^[73]^. Consistent findings were observed *in vitro*: BMSC-Exos co-cultured with CD4^+^ T cells from DED patients downregulated IL-17 and IL-22 expression while upregulating IL-4, IL-10, and TGF-β1, leading to a reduced Th17 cell proportion and an expanded Treg population^[73]^. The therapeutic potential of MSC-EVs extends to Sjögren’s syndrome, a chronic inflammatory autoimmune disease characterized by xerostomia and keratoconjunctivitis sicca (dry eyes)^[138]^. In mice with experimental Sjögren’s syndrome, intraorbital injection of MSC-EVs attenuated the infiltration of T cells in the lacrimal glands. A strong positive correlation was found between the levels of key mediators TGF-β1, pentraxin 3 (PTX3), let-7b-5p, and miR-21-5p in MSC-EVs and their immunosuppressive function^[106]^. Furthermore, in both non-obese diabetic mouse models and *in vitro* cultures of peripheral blood mononuclear cells from Sjögren’s syndrome patients, labial gland-derived MSC exosomes (LGMSC-Exos) suppressed Th17 cell differentiation and promoted Treg induction, thereby restoring the Th17/Treg balance. This immunomodulatory effect was accompanied by reduced production of IL-17, IFN-γ, and IL-6, alongside increased production of TGF-β and IL-10 by T cells, indicating partial suppression of inflammatory responses^[139]^. In patients with primary Sjögren’s syndrome, human UCMSC-Exos suppressed excessive proliferation and apoptosis of CD4^+^ T cells and blocked cell cycle progression in the G0/G1 phase, thereby inhibiting S phase entry. Furthermore, UCMSC-Exos rebalanced the Th17/Treg ratio by reducing the proportion of Th17 cells and elevating that of Tregs, accompanied by a reduction in the levels of IFN-γ, TNF-α, IL-6, IL-17A, and IL-17F and elevation in the levels of IL-10 and TGF-β. Mechanistically, UCMSC-Exos regulate CD4^+^ T cell immunity in primary Sjögren’s syndrome by reducing the elevated autophagic flux in peripheral blood CD4^+^ T cells^[140]^. Topical treatment with MSC-EVs in DED mice considerably decreased the Th17 cell population in the draining lymph nodes by inhibiting DC activation-mediated Th17 immune responses^[134]^.

MSC-EVs can also suppress B lymphocyte-mediated immune responses. When peripheral blood mononuclear cells from primary Sjögren’s syndrome patients were co-cultured with LGMSC-Exos, the proportion of CD19+CD20-CD24+CD38+ plasma cells significantly decreased. The underlying mechanism involves exosomal miR-125b from LGMSC-Exos binding to PR domain zinc finger protein 1 (PRDM1) mRNA and suppressing its translation, thereby inhibiting plasma cell differentiation. The miR-125b contained within LGMSC-Exos is essential for modulating autoantibody production and suppressing B cell-mediated autoimmune responses in primary Sjögren’s syndrome^[141]^. PRDM1 (also designated B lymphocyte-induced maturation protein-1, BLIMP-1) is a transcription factor indispensable for directing B cell differentiation into plasma cells capable of antibody production.

#### Tissue reparative and regenerative effects of MSC-EVs in the treatment of DED

Beyond their anti-inflammatory and immunomodulatory functions, MSC-EVs can exert reparative and regenerative functions by inhibiting apoptosis, promoting cell proliferation, and regulating angiogenesis through the bioactive molecules they carry^[102,123-125,129,135,136]^. Extensive studies^[21,73,106,127-132,134,135,137]^ across murine, rabbit, and human models of DED and Sjögren’s syndrome-related ocular damage have demonstrated the comprehensive therapeutic benefits of MSC-EV treatment. MSC-EVs can restore ocular surface homeostasis, thereby providing protection against desiccating and hyperosmotic stress. Key improvements include enhanced tear film stability (evidenced by prolonged tear film breakup time and increased tear secretion), reduced ocular surface disease index (OSDI) scores, and significant alleviation of corneal epithelial damage (evidenced by fluorescein staining) and conjunctival hyperemia. Moreover, substantial mitigation of corneal damage, together with improved epithelial integrity have been consistently observed. These effects are characterized by a well-organized arrangement of corneal epithelial cells, stromal cells, nerves, and endothelial cells, as well as increased numbers of corneal epithelial layers, thickening of the corneal and conjunctival epithelia, and active proliferation of corneal stromal cells. Concurrently, MSC-EVs effectively suppressed apoptosis in both corneal stromal cells and conjunctival epithelial cells, while promoting conjunctival goblet cell proliferation and upregulating the expression of their specific proteins, mucin-1 (MUC-1) and mucin-5AC (MUC-5AC). By specifically targeting myofibroblast activation and differentiation, UCMSC-Exos exhibit anti-fibrotic potential. The observed reduction in myofibroblast formation indicates their potential to prevent corneal fibrotic changes that lead to scarring and impaired vision^[129]^.

MSC-EVs promote corneal wound healing and repair through the action of their diverse cargo of growth factors. For example, ADMSC-Exos enhance the growth and plasticity of corneal stromal cells by inhibiting MMPs, upregulating ECM-related proteins including collagens and fibronectin, and thereby promoting ECM synthesis^[142]^. The inhibition of MMP activation effectively prevents collagen degradation and matrix thinning, thereby indirectly maintaining matrix homeostasis^[129,142]^. Furthermore, in mouse models of DED, treatment with human UCMSC-EVs altered the cytokine profile in tears, elevating epidermal growth factor (EGF) concentrations while reducing vascular endothelial growth factor (VEGF) levels. Notably, in a rat DED model, administration of UCMSC-Exos significantly reduced the length and branching of corneal neovessels. These consistent anti-angiogenic effects across species suggest the preseof specific anti-angiogenic factors or regulatory molecules within human UC-EVs that can modulate genes involved in angiogenesis. Thus, human UCMSC-EVs demonstrate potential to simultaneously promote corneal regeneration and suppress pathological neovascularization^[127,129]^. Treatment of Sjögren’s syndrome-associated DED with topical ophthalmic human WJMSC-Exos led to the upregulation of genes encoding multifunctional healing-promoting peptides, including lactoferrin, EGF, and thrombospondin-1 (TSP-1)^[128]^. EGF, a potent polypeptide mitogen, is present in both the tear film and serum. Synthesized and released by the lacrimal gland, it plays a key role in maintaining corneal epithelial integrity and facilitating wound healing^[128]^. Lactoferrin constitutes approximately 25% of total tear proteins and alleviates dryness in DED patients by reducing oxidative stress and inflammation^[62]^. Oral administration of enteric-coated lactoferrin capsules at a daily dose of 270 mg has also demonstrated efficacy, promoting goblet cell secretion and extending tear film break-up time^[143]^. TSP-1 is a key activator of latent TGF-β, thereby enabling the immunomodulatory and wound-healing activities of TGF-β. In addition, TSP-1 is also a potent endogenous inhibitor of angiogenesis. Although this anti-angiogenic effect has not been explicitly demonstrated in the cornea, TSP-1 is known to exert its anti-angiogenic effects through other critical signaling pathways^[144]^.

Current research on the mechanisms of MSC-EVs in the treatment of DED largely focuses on their anti-inflammatory and immunomodulatory effects, whereas their effects on tissue repair and regeneration remain insufficiently explored. In contrast, these regenerative functions have been more widely studied in other non-DED corneal disorders. Treatment with human UCMSC-sEVs has been demonstrated to remarkably improve the proliferation and migration of corneal epithelial cells *in vitro*, as well as corneal epithelial wound healing *in vivo*, by delivering miR-21, which suppresses PTEN and consequently activates the PI3K/Akt signaling pathway^[145]^. Similarly, EVs from bone marrow MSCs (BMMSC-EVs) promote corneal epithelial repair by enhancing cell proliferation and inhibiting apoptosis through the downregulation of pro-apoptotic genes encoding BCL-2-associated agonist of cell death (BAD), BCL-2-associated X-protein (BAX), P53, and caspase 3 and the concurrent upregulation of the anti-apoptotic gene encoding B-cell lymphoma 2 (BCL-2)^[146,147]^. ADMSC-Exos facilitate corneal endothelial wound healing and promote the migration of corneal epithelial cells both *in vitro* and *in vivo* through a dose-dependent induction of the G0/G1-to-S-phase cell cycle shift, inhibition of ROS-mediated senescence, and suppression of autophagy - thereby inhibiting epithelial-mesenchymal transition while restoring mitochondrial function and promoting corneal epithelial regeneration^[148]^. In addition, human BMMSC-EVs enhance regeneration of mouse trigeminal ganglion neurons. Notably, EVs derived from three-dimensional (3D) culture systems significantly promote neurite elongation, branching, and complexity compared with those derived from two-dimensional (2D) cultures^[122]^.

**Figure 4:**
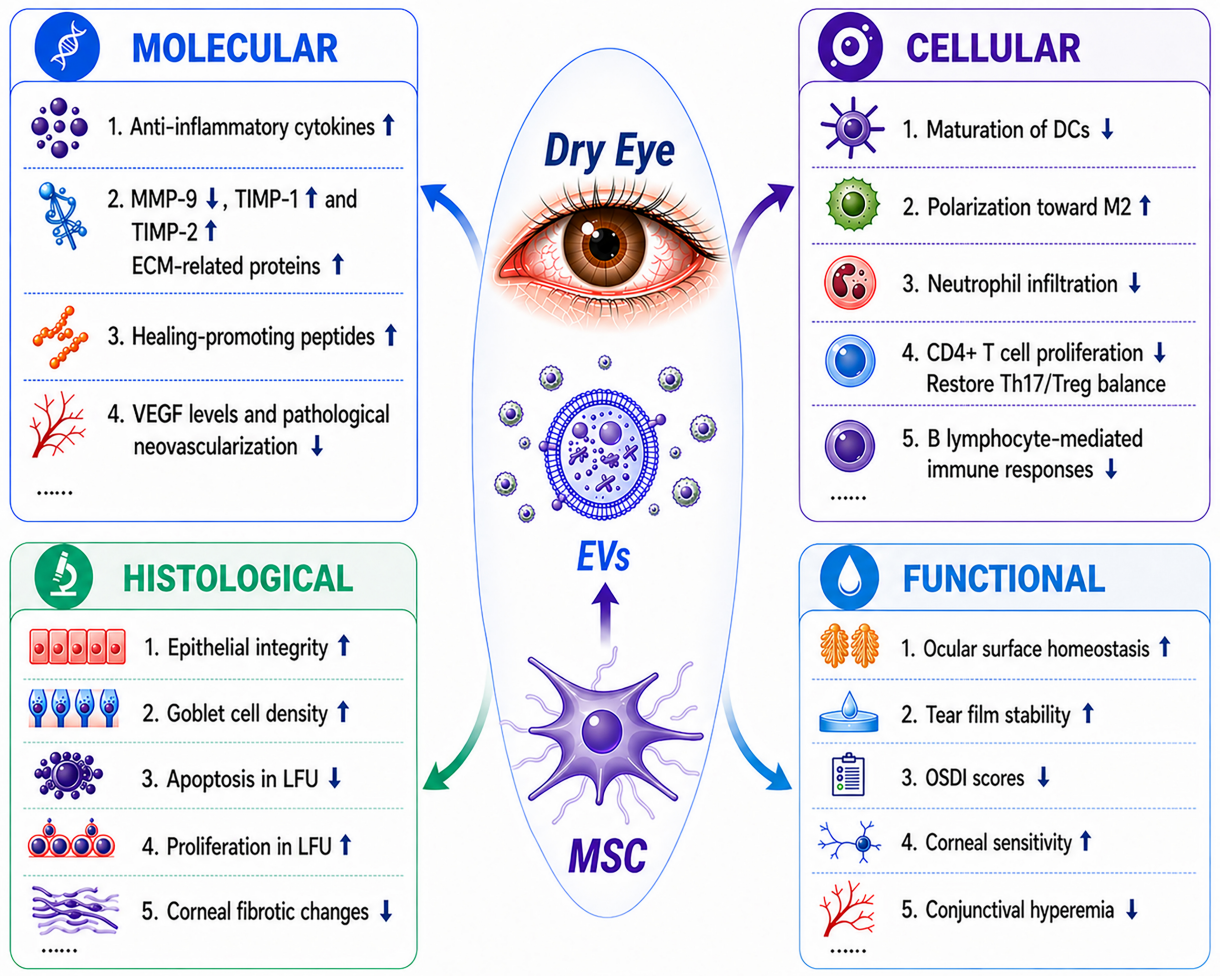
Multilevel therapeutic effects of MSC-EVs in dry eye disease: molecular, cellular, histological, and functional perspectives. DCs: Dendritic cells; ECM: extracellular matrix; EVs: extracellular vesicles; LFU: lacrimal functional unit; MMP-9: matrix metalloproteinase-9; MSC: mesenchymal stem cell; OSDI: ocular surface disease index; TIMP-1: tissue inhibitor of metalloproteinases-1; TIMP-2: tissue inhibitor of metalloproteinases-2; VEGF: vascular endothelial growth factor.

In summary, leveraging their anti-inflammatory, immunomodulatory, reparative, and regenerative properties, MSC-EVs demonstrate considerable therapeutic potential for DED at the molecular, cellular, histological, and functional levels [Figure 4].

Table 1 summarizes *in vivo* studies using MSC-EVs in dry eye and related diseases. The study by Habibi *et al*. reported a randomized controlled trial in patients with primary Sjögren’s syndrome that employed a contralateral-eye design and assessed tear protein biomarkers (e.g., IL-6, MMP-9, and EGF)^[128]^. However, the small sample size (*n* = 8) and the lack of characterization of specific exosomal cargo limit the strength and generalizability of its conclusions. Mechanistic insights are strengthened by the study of Zhou *et al*., which employed both loss- and gain-of-function approaches to support a causal role for miR-204^[21]^. In contrast, most other studies rely primarily on inhibitor-based or correlative analyses, resulting in weaker causal inference. From a methodological perspective, the study by Kim *et al*. represents a rigorous approach, systematically comparing early-passage (15 population doublings, PD15) *vs*. late-passage (PD40) MSCs, and 2D *vs*. 3D culture conditions, while integrating proteomics and miRNA sequencing to evaluate EV function^[106]^. These findings provide a useful framework for EV production and quality control, although human validation remains lacking. Additionally, the study by Zhao *et al*. established a mechanistic-phenotypic association by implicating the miR-21-5p/TLR4/MyD88/NF-κB axis in restoring Treg/Th17 balance^[73]^, although this was demonstrated in a benzalkonium chloride-induced chemical injury model that may not fully recapitulate autoimmune dry eye pathology.

**Table 1:** Summary of *in vivo* studies on MSC-EVs in dry eye and related diseases

**No.**	**Disease**	**Subjects**	**Source of MSCs**	**EV isolation method**	**Dosage regimen**	**Administration route**	**Key cargo**	**Possible target**	**Major outcomes**	**Ref.**
1	DED (Desiccation-induced)	Female C57BL/6 mice	Human UCMSCs	UC	0.5-5.0 mg/mL 5 μL/eye 4 times/day for 21 days	Topical eye drops	MiRNAs (miR-125b, let-7b, miR-6873, *etc*.)	IRAK1/TAB2/NF-κB pathway	Increased tear volume, reduced corneal staining, decreased inflammatory cytokines, increased goblet cell density, reduced apoptosis and CD4+ T cell infiltration	[127]
2	DED (BAC-induced)	Male BALB/c mice	BMSCs	Precipitation-based exosome isolation	50 μg/mouse every other day for 14 consecutive days	Tail vein injection	miR-21-5p	TLR4/MyD88/ NF-κB pathway	Increased tear secretion, restored corneal epithelium, increased goblet cell density, rebalanced Treg/Th17 ratio (decreased Th17, increased Treg), reduced IL-17 and IL-22, increased IL-4, IL-10, TGF-β1	[73]
3	SS-associated DED	NOD.B10.H2^b^ mice	Human MSCs	SEC or UC	5 × 10^8^ particles in 20 μL of PBS	Intraorbital lacrimal gland injection	TGF-β1, PTX3, let-7b-5p, miR-21-5p	TCR signaling/ TLR4/NF-κB pathway	Improved corneal epithelial integrity, increased tear production, reduced inflammatory cytokines (TNF-α, IL-1β, IFN-γ), increased conjunctival goblet cells, reduced T cell infiltration in lacrimal gland	[106]
4	DED BAC-induced	Male BALB/c mice	Mouse ADMSC	N/A	1 × 10^6^ vg/mouse^*^	Intraconjunctival injection	miR-223-3p	Fbxw7 inhibition suppressing hyperosmolarity-induced inflammation	Reduced fluorescein staining, increased tear volume and BUT, decreased pro-inflammatory cytokines (IL-6, TNF-α, IL-1β, IL-17, CCL2, CXCL1), increased goblet cells, reduced apoptosis, improved corneal/conjunctival histology	[130]
5	DED Scop-induced	Male BALB/c mice	Mouse ADMSC	N/A	1 × 10^6^ vg/mouse^*^	Intraconjunctival injection	miR-223-3p	Same as above	Improved fluorescein staining, tear volume, BUT, and reduced inflammation	[130]
6	DED Desiccation-induced	Female C57BL/6 mice	Human ADMSCs	UC Amico Exoquick	5 μL of 1 μg/μL (5 μg/eye), 4 times daily for 5 days	Topical eye drops	EVs	NLRP3 inflammasome (NLRP3/ASC/caspase-1) and IL-1β	Reduced corneal fluorescein staining, increased tear production, increased conjunctival goblet cells and Muc-5AC/Muc-1 expression, suppressed apoptosis (γ-H2AX), inhibited NLRP3 inflammasome activation and IL-1β maturation	[131]
7	DED BAC-induced	Male C57BL/6 mice	Mouse ADMSCs	dUC	5 μL/eye (12.5, 25, 50 mg/mL), 3 times daily for 7 days	Topical eye drops	Exosomes	NLRP3 inflammasome (NLRP3/caspase-1) and downstream IL-1β/IL-18, PI3K/NF-κB pathway	Reduced corneal fluorescein staining, increased tear volume and BUT, restored goblet cells, inhibited apoptosis (TUNEL, caspase-3), decreased pro-inflammatory cytokines (IL-1β, IL-6, IL-1α, IFN-γ, TNF-α), increased IL-10, suppressed NLRP3 inflammasome activation and PI3K/NF-κB signaling	[132]
8	SS-related DED	Clinical trial (Phase I/II)	Human WJMSCs	dUC	10 μg/drop (concentration 100 μg/mL), twice daily for 2 weeks	Topical eye drops	Exosomes	Not specified	Significant improvement in OSDI, tear secretion, BUT corneal fluorescein staining and conjunctival redness; increased tear EGF and THBS1 gene expression; decreased tear IL-6 and MMP-9; no local or systemic adverse events. Therapeutic effect partially diminished by 12 weeks after treatment cessation	[128]
9	Severe DED (Aqueous-deficient)	Female sprague dawley rats	UCMSCs	TFF	20-30 µL/eye (115 × 10^8^ particles/0.5 mL), twice daily for 56 days (starting 48 h post-surgery)	Topical eye drops	Exosomes	Inflammatory cytokines (IL-1β, TNF-α, IL-10), apoptosis, myofibroblast (α-SMA), neutrophil infiltration	Reduced corneal neovascularization, decreased fluorescein staining area during first month, corneal epithelial thickness closer to normal, reduced apoptosis, decreased neutrophil infiltration, lower α-SMA expression, downregulated IL-1β and TNF-α, upregulated IL-10 at day 7	[129]
10	GVHD-associated DED	C57BL/6J mice; NCG-GVHD mice	Mouse BMMSCs	UC	5 μL/drop (2.5 × 10^10^ particles/mL), twice daily for 7 days	Topical eye drops	miR-204-5p	IL-6R/IL-6/Stat3 pathway	Reduced corneal fluorescein staining, increased tear volume, suppressed corneal epithelial defects and inflammation, decreased M1 macrophages, increased M2 macrophages, improved ocular surface structure	[21]
11	Refractory GVHD-associated DED	Clinical trial (phase not specified)	Human UCMSCs	UC	10 μg/50 μL per drop, four times daily for 2 weeks	Topical eye drops	miR-204-5p	IL-6R/IL-6/Stat3 pathway	Significant reduction in fluorescein staining scores, increased tear-film BUT, increased tear secretion, lower OSDI scores, no adverse events; symptoms relieved (sting, burn, redness)	[21]
12	SS-related DED (early stage)	Female New Zealand	Human hUCMSCs	dUC	30 μg per dose, on days 1, 3, 5, 7, 9 after adoptive transfer (5 doses total)	Subconjunctival injection	miR-100-5p	M2 macrophage polarization, Treg induction	Reduced corneal fluorescein staining, increased tear BUT and tear production, decreased lymphocyte infiltration in lacrimal glands and conjunctiva, decreased M1 markers (NOS2, IRF5, TNF-α, IL-1β, IL-6), increased M2 markers (Arg1, CD206, KLF4, IL-10, TGF-β), increased CD4+Foxp3+ Tregs in lacrimal glands	[135]
13	SS-related DED (developed stage)	Female New Zealand	Human UCMSCs	dUC	30 μg per dose, every other day for 5 doses (2 weeks after transfer)	Subconjunctival injection	miR-100-5p	M2 macrophage polarization, Treg induction	Improved clinical signs (fluorescein staining, tear BUT, tear production) from week 4 to week 8; reduced lymphocyte infiltration in lacrimal glands and conjunctiva; similar molecular changes as preventive group (M2 skewing, Treg increase)	[135]
14	SS	Female NOD mice	LGMSCs	dUC	50 μg per mouse, on alternate days for 2 weeks	Intravenous (tail vein)	exosomes	Th17/Treg balance; IL-17, IL-6, TGF-β	Increased saliva flow rate, reduced lymphocytic infiltration in salivary glands, decreased Th17 cell proportion, increased Treg cell proportion in spleen, reduced serum IL-17 and IL-6, increased serum TGF-β and IL-10	[139]
15	pSS	Female NOD mice	LGMSCs	dUC	50 μg/200 μL PBS/mouse, on alternate days for 14 days	Intravenous (tail vein)	miR-125b-5p	PRDM1 (Blimp1)	Reduced lymphocytic infiltration in salivary glands (smaller focus area), restored saliva flow rate, decreased proportion of CD19+CD138+ plasma cells in spleen (no change in total B cells or memory B cells)	[141]

^*^The dose “1 × 10^6^ vg/mouse” corresponds to a lentiviral vector (used to overexpress or knock down miR-223-3p), not extracellular vesicles. Vg is a standard unit for lentiviral quantification. All other rows list extracellular vesicle doses. ADMSCs: Adipose-derived mesenchymal stem cells; α-SMA: alpha-smooth muscle actin; Arg1: arginase 1; ASC: apoptosis-associated speck-like protein containing a CARD; BAC: benzalkonium chloride; Blimp1: B-lymphocyte-induced maturation protein 1 (same as PRDM1); BMSCs: bone marrow mesenchymal stem cells; BUT: break-up time; CCL2: chemokine (C-C motif) ligand 2; CD206: cluster of differentiation 206 (mannose receptor); CXCL1: C-X-C motif chemokine ligand 1; DED: dry eye disease; dUC: differential ultracentrifugation; EGF: epidermal growth factor; EVs: extracellular vesicles; Fbxw7: F-box and WD repeat domain-containing 7; FOXP3: forkhead box P3; GVHD: graft-versus-host disease; IFN-γ: interferon gamma; IL: interleukin; IL-6R: interleukin-6 receptor; IRAK1: IL-1 receptor-associated kinase 1; IRF5: interferon regulatory factor 5; KLF4: Kruppel-like factor 4; LGMSCs: labial gland mesenchymal stem cells; miRNA: microRNA; MMP-9: matrix metalloproteinase 9; MSCs: mesenchymal stem cells; Muc-1: mucin 1; Muc-5AC: mucin 5AC; MyD88: myeloid differentiation primary response 88; NCG: NOD/ShiLtJGpt-Prkdcem26Cd52Il2rgem26Cd22/Gpt (a mouse strain); NF-κB: nuclear factor kappa-light-chain-enhancer of activated B cells; NLRP3: NLR family pyrin domain containing 3; NOD: non-obese diabetic (mouse strain); NOS2: nitric oxide synthase 2 (iNOS); OSDI: ocular surface disease index; PBS: phosphate-buffered saline; PI3K: phosphoinositide 3-kinase; PRDM1: PR domain zinc finger protein 1; pSS: primary Sjögren’s syndrome; PTX3: pentraxin 3; Scop: scopolamine; SEC: size-exclusion chromatography; SS: Sjögren’s syndrome; Stat3: signal transducer and activator of transcription 3; TAB2: TGF-β-activated kinase 1 binding protein 2; TCR: T cell receptor; TFF: tangential flow filtration; TGF-β1: transforming growth factor beta 1; THBS1: thrombospondin-1; TLR4: Toll-like receptor 4; TNF-α: tumor necrosis factor alpha; Treg: regulatory T cell; TUNEL: terminal deoxynucleotidyl transferase dUTP nick end labeling; UC: ultracentrifugation; UCMSCs: umbilical cord mesenchymal stem cells; vg: viral genomes; WJMSCs: Wharton’s jelly mesenchymal stem cells; γ-H2AX: phosphorylated histone variant H2AX.

Many studies have utilized topical administration of MSC-EVs as eye drops^[21,127-129,131,132]^, providing a non-invasive and convenient route that restores ocular surface immune homeostasis and mitigates inflammatory damage, highlighting strong translational potential.

Common limitations across studies include non-standardized dosing units, short follow-up (≤ 56 days in animals; ≤ 3 months in humans), and heterogeneity in MSC sources, isolation methods, and investigated miRNAs. Clinical validation remains limited, with only a few human studies, and most investigations remain preclinical, requiring further confirmation in well-designed clinical trials.

### Treatment of DED with engineered MSC-EVs

Although EVs naturally cross biological barriers and deliver bioactive cargo due to their lipid bilayer structure, nanoscale size, and tropism inherited from their parental cells, several challenges remain in ocular applications. These include the anatomical and physiological barriers and the rapid clearance of topically applied drugs by the tear film, which limit therapeutic retention. To address these challenges, diverse engineering strategies have been developed, such as cargo modification, surface functionalization, hybrid system construction, and genetic engineering, to enhance EV performance^[149-153]^. Despite these advances, studies specifically focusing on engineered MSC-EVs in DED remain relatively limited^[154,155]^. Therefore, broader EV engineering strategies and associated ocular applications should be considered to improve MSC-EV-based therapy for DED.

#### Cargo engineering of MSC-EVs: enhancing therapeutic functionality

Current studies on engineered MSC-EVs in DED primarily focus on integration of functional cargo, particularly targeting the core pathological axis of “inflammation-ROS-injury”. Besides nanoparticle-based modification, cargo engineering can be achieved through techniques such as electroporation, sonication, and donor cell preconditioning to load small molecules, proteins, or nucleic acids^[149,150,152,156,157]^.

A representative example of cargo engineering is MSCExo-Ce, a nanoparticle eye drop generated using *in situ* crystallization of cerium oxide nanocrystals onto MSC-derived exosomes^[154]^. This system synergistically integrates the intrinsic anti-inflammatory and pro-regenerative properties of MSC-EVs with the strong ROS-scavenging capacity of cerium oxide. Experimental results demonstrated that MSCExo-Ce significantly promotes corneal epithelial cell migration and efficiently eliminates intracellular ROS *in vitro*. In a benzalkonium chloride-induced DED model, MSCExo-Ce markedly improved tear secretion and reduced corneal epithelial damage. It also suppressed IL-1β expression and inhibited apoptosis. Overall, it exhibited superior therapeutic efficacy compared with unmodified MSC-EVs, while maintaining excellent biocompatibility.

Another example is mExo@AA, developed by depositing ascorbic acid-reduced gold nanoparticles onto MSC-EV membranes^[155]^. This engineered system preserves the native properties of EVs while adding better antioxidative and immunomodulatory functions. *In vitro*, mExo@AA accelerated epithelial wound healing and scavenged ROS. It also promoted M2 macrophage polarization and reduced pro-inflammatory cytokines like IL-6 and IL-1β. *In vivo*, it outperformed both EVs and ascorbic acid alone, showing synergistic effects on corneal repair, inflammation suppression, and tear restoration. Only the combined system completely cleared ROS - a clear demonstration of how cargo-engineered MSC-EVs can serve as multifunctional platforms.

#### Hybrid and non-MSC EV engineering: complementary strategies for precision delivery

Given the limited number of MSC-EV-focused studies, engineering strategies derived from non-MSC EV systems provide important complementary insights, particularly in improving targeting specificity and delivery efficiency. Hybrid engineering approaches, including membrane fusion, extrusion, and chemical conjugation, have also been explored to further enhance EV targeting and cargo delivery^[149,150,157,158]^.

For example, a hybrid exosome vehicle was constructed by fusing small interfering RNA (siRNA)-loaded liposomes with corneal epithelial cell-derived EVs via DNA zipper-mediated membrane fusion^[158]^. This design enables precise delivery of anti-NF-kappa-B inhibitor zeta gene (NFKBIZ) siRNA to corneal tissues, effectively silencing inflammatory signaling pathways. The hybrid exosome vehicle system reduced pro-inflammatory cytokine production and reprogrammed macrophages from an M1 to an M2 phenotype. It also decreased apoptosis and enhanced epithelial proliferation. *In vivo*, it significantly improved tear secretion and restored corneal integrity without detectable toxicity. Such hybrid engineering strategies demonstrate how EVs can be transformed from natural carriers into programmable delivery systems, offering valuable design principles that can be readily adapted to MSC-EV-based platforms.

#### Biomaterial-assisted EV delivery: improving retention and bioavailability

Biomaterial-based delivery systems, particularly hydrogels, help engineered MSC-EVs overcome rapid clearance and limited ocular retention^[159,160]^. Beyond hydrogels, alternative platforms such as nanoparticles, microneedles, and contact lens-based delivery systems have also been explored to improve ocular drug retention and controlled release^[161-163]^.

A thermosensitive chitosan-based hydrogel loaded with induced pluripotent stem cell-derived mesenchymal stem cells (iPSC-MSCs) enables sustained release and prolonged ocular surface residence^[159]^. The system delivers miR-432-5p, which suppresses collagen production by targeting translocation-associated membrane protein 2 (TRAM2), thereby reducing fibrosis and promoting corneal epithelial and stromal repair. This approach improves corneal transparency while inhibiting neovascularization. Thermosensitive hydrogels effectively reduce drug loss caused by nasolacrimal drainage, significantly enhancing local bioavailability^[164]^.

Exos-miRNA-24-3p combined with a di(ethylene glycol) methyl ether methacrylate (DEGMA)-modified hyaluronic acid hydrogel enables sustained ocular delivery, accelerating epithelial wound closure, reducing stromal fibrosis, and suppressing macrophage activation^[160]^. This system markedly improves corneal repair outcomes over extended treatment periods, demonstrating that hydrogel-assisted platforms can substantially enhance the clinical translation potential of engineered MSC-EVs by improving drug retention and delivery efficiency.

#### Genetic engineering of EVs: toward targeted immunomodulation

Beyond physicochemical and delivery-based modifications, genetic engineering of EV cargo is an emerging strategy for targeting immunoregulation. Approaches include miRNA enrichment, siRNA loading, and clustered regularly interspaced short palindromic repeats (CRISPR)-based modifications to precisely modulate disease-related pathways^[149,150,157,158]^. EVs derived from LGMSCs and loaded with miRNA let-7f-5p showed significant therapeutic efficacy in a Sjögren’s syndrome model^[165]^. Mechanistically, these engineered EVs targeted the 3’ untranslated region of RAR-related orphan receptor C (RORC), suppressing the RORC/IL-17A signaling axis and decreasing IL-17A production, which restored Th17/Treg immune balance. Therefore, engineered EVs can modulate disease-specific immune pathways at the molecular level, a precision medicine strategy for ocular surface inflammation.

Direct evidence for engineered MSC-EVs in DED remains limited. However, existing studies demonstrate that cargo modification, surface functionalization, hybrid systems, and genetic engineering can transform MSC-EVs into multifunctional, programmable platforms. The integration of insights from broader EV engineering strategies not only helps bridge current evidence gaps, but also highlights pathways for advancing MSC-EV-based therapies. As engineering technologies evolve, MSC-EVs are expected to transition from passive carriers to precisely controllable, mechanism-driven systems, thereby shifting DED management from symptom relief to true disease modification.

### From lab to clinic: 3D ocular surface models and MSC-EVs for DED

#### 3D ocular surface models: preclinical progress and translational hurdles

Although animal models of DED have yielded promising results, translating these findings is not straightforward due to major differences in anatomy, immunity, and physiology between animal and human eyes. For example, the human eyeball has a volume of about 6-7 mL, while the conjunctival sac, the main site where eye drops pool after topical application, holds only about 30 µL. In rodents (mice and rats), by contrast, the eyeball volume is merely 0.15-0.2 mL, and their conjunctival sac is proportionally even smaller^[151,166]^. To circumvent these limitations, researchers in recent years have turned to 3D ocular surface models. Built from human cells, these *in vitro* systems recapitulate human ocular physiology faithfully at the cellular and microtissue level, thus helping to overcome the interspecies gaps^[167-170]^.

Recent work has shown that 3D ocular surface models can recreate the native structure of the cornea, lacrimal gland, and meibomian gland. As a result, these models offer more physiologically relevant platforms for studying DED than conventional 2D cultures or animal models. Consider long-term human corneal epithelial organoids derived from adult stem cells: they form a stratified epithelium, express functional markers like the ΔNp63α isoform of p63, keratin 12 (KRT12), and MUC1, and under hyperosmotic stress they upregulate pro-inflammatory cytokines (NF-κB1, IL-6, IL-8) while losing stemness, closely mimicking key aspects of DED pathophysiology^[170]^. Human lacrimal gland organoids, generated from pluripotent stem cells or primary tissue, similarly keep their acinar and ductal features, like expression of aquaporin 5 (AQP5) and lysozyme (LYZ). They respond to cholinergic stimulation with calcium influx and LYZ release, and can serve as models for both Sjögren’s syndrome and age-related lacrimal gland hypofunction^[171-173]^. For meibomian gland dysfunction, the leading cause of evaporative DED, both mouse and human meibomian gland organoids maintain holocrine lipid secretion. Studies using these organoids have also revealed that fibroblast growth factor (FGF) 10 is essential for their expansion and can prevent all-trans retinoic acid-induced meibomian gland atrophy *in vivo*^[174]^. Meanwhile, microfluidic cornea-on-a-chip devices integrate an air-liquid interface, tear flow, and blinking mechanics, which allows real-time assessment of barrier function and drug permeability under shear-stress conditions relevant to DED^[168,175]^.

Researchers are increasingly using these 3D *ex vivo* models to screen drugs, investigate disease mechanisms, and develop regenerative therapies for DED. Corneal epithelial organoids have been successfully employed to evaluate clinically relevant DED drugs. For example, cyclosporine A suppressed inflammation, diquafosol improved cell viability, and basic FGF (bFGF) promoted proliferation, showing concordance with known clinical responses^[170]^. Dome-shaped 3D corneal constructs, which have adjustable curvature, have allowed researchers to model ultraviolet B (UVB)-induced photokeratitis and to confirm the protective effect of eye drops containing bFGF^[176]^. In addition, lacrimal gland and meibomian gland organoids are being explored for cell-based repair applications. Orthotopic transplantation of human meibomian organoids into immunodeficient mice resulted in engraftment, expression of mature meibocyte markers such as peroxisome proliferator-activated receptor gamma (PPARγ), and lipid production^[174]^. Senescence-associated lacrimal gland organoids generated via magnetic 3D bioprinting showed that high mobility group box-1 (HMGB1) Box A gene therapy could prevent etoposide-induced apoptosis, restore ATP levels, and preserve secretory function^[177]^. Altogether, these 3D ocular surface models bridge the gap between simple cell cultures and animal studies, offering predictive, human-relevant platforms for understanding DED pathogenesis and accelerating the development of novel therapeutics, including regenerative medicine approaches.

Importantly, the successes described above, including *in vitro* drug evaluations and *in vivo* animal transplantation studies, remain largely preclinical. Consequently, the potential of 3D ocular surface models has yet to bridge the gap from bench to bedside. Ma *et al*. observed that corneal organoid transplantation in humans has so far only been achieved using tissue fragments for ocular surface reconstruction. Transplanting a complete, intact corneal organoid is still only possible in animals^[178]^. Similarly, current cornea-chip models still fall short of fully reproducing the cornea’s natural curvature, basement membrane, or innervation^[169]^. Lacrimal gland organoids, for their part, continue to face hurdles in becoming vascularized, innervated, and functionally mature over the long term^[179]^. Consequently, while these models hold promise for future clinical applications, robust human trials evaluating organoid- or chip-based therapies for DED have not yet been reported.

#### Clinical trials of MSC-EVs for DED: from bench to bedside

While organoid- and chip-based therapies remain preclinical, MSC-EVs have already entered clinical trials for DED. Inspired by the promising therapeutic effects of MSC-EVs observed in preclinical studies, as of June 2025, eight clinical trials investigating MSC-EVs for DED have been registered across ClinicalTrials.gov, ChiCTR, and the WHO ICTRP platforms [Table 2].

**Table 2:** Summary of clinical trials of MSC-EVs for DED

**No.**	**Trial ID**	**Disease type**	**Inclusion criteria (abridged)**	**EV source**	**Dosage and administration**	**Phase/Status**	**Registration date**	**Country**
1	ChiCTR2500102146	Severe refractory dry eye	Age 18-70; diagnosed with severe dry eye; discontinued other dry eye medications for > 1 week; no participation in other drug trials within 3 months	Human umbilical cord MSC apoptotic vesicles	Eye drops (dose not specified)	Pre-registration/Not yet started	2025/05/09	China
2	ChiCTR2500099859	SS-related dry eye	Age 18-70; confirmed SS; no response to conventional treatment for ≥ 3 months	Human umbilical cord MSC	Eye drops (dose not specified)	Not yet started	2025/03/31	China
3	IRCT20170211032503N2	Severe dry eye	Severe dry eye symptoms; no response to conventional treatment; Schirmer test ≤ 10 mm/5 min	Not specified	Eye drops (dose not specified)	Early Phase 1/Not yet started	2024/02/20	Iran
4	IRCT20231022059814N1	SS-related dry eye	Age 18-70; moderate-to-severe, resistant to conventional treatment	Limbal MSC	Eye drops (10 μg/drop)	Phase 1/Recruiting	2023/11/11	Iran
5	NCT05738629	Dry eye associated with post-refractive surgery or blepharospasm	Confirmed dry eye; no response to artificial tears for ≥ 3 months; Schirmer ≤ 10 mm/5 min	Pluripotent stem cell MSC	Eye drops (0.125 mL/eye/time, 4 times/day for 12 weeks)	Phase 1/2/Not yet started	2023/02/13	China
6	IRCT20211102052948N1	SS-related dry eye	Age 35-45; clinical/serologically confirmed SS; no response to conventional treatment	Wharton’s jelly MSC	Eye drops (10 μg/time, twice daily for 2 weeks)	Phase 1/Recruiting	2022/04/20	Iran
7	NCT04213248	cGVHD-related dry eye	Diagnosed with cGVHD with significant dry eye; no response to artificial tears; Schirmer ≤ 10 mm/5 min	Umbilical cord MSC	Eye drops (10 μg/drop, 4 times/day, 1 drop each time for 14 days)	Phase 1/2/Recruiting	2020/02/21	China
8	ChiCTR2000031188	Dry eye (especially moderate-to-severe with goblet cell reduction)	Age 20-60; meets clinical dry eye diagnosis; able to follow up on time	Human umbilical cord MSC	Eye drops (dose not specified)	Pre-registration/Status unclear	2020/03/23	China

Data collected from: https://clinicaltrials.gov/, http://www.chictr.org.cn/ and https://trialsearch.who.int/ websites (accessed on 14 June 2025). cGVHD: Chronic graft-versus-host disease; DED: dry eye disease; EVs: extracellular vesicles; MSC: mesenchymal stem cells; SS: Sjögren’s syndrome.

Obvious heterogeneity exists among these eight clinical trials regarding patient enrollment criteria, dosing regimens, cell sources, and reported outcomes.

(1) Heterogeneity in patient enrollment criteria. Age ranges vary from 20-60 years to 18-70 years. Disease subtypes include Sjögren’s syndrome, chronic graft-versus-host disease, post-refractive surgery, and non-specific dry eye. Objective severity thresholds, such as a Schirmer test score ≤ 10 mm/5 min, are employed as inclusion criteria in several studies.

(2) Heterogeneity in dosing and administration. Dosing strategies remain unstandardized. Three trials adopt a fixed concentration of 10 μg/drop, whereas one trial specifies a fixed volume of 0.125 mL/eye per dose. Administration frequency ranges from twice daily to four times daily. These discrepancies underscore the need for further dose-finding and regimen-optimization studies.

(3) Efficacy and results. To date, only two trials have published peer-reviewed efficacy data: one demonstrated statistically significant improvements in OSDI score, Schirmer I test, and tear break-up time (IRCT20211102052948N1)^[128]^; the other reported clinical benefit in chronic graft-versus-host disease -associated dry eye, with mechanistic evidence implicating miR-204 cargo (NCT04213248)^[21]^.

(4) Cell source. Most trials utilize UCMSCs. One trial employs limbal stem cells, and another uses pluripotent stem cell-derived MSCs. The impact of differing cell sources on clinical outcomes remains to be elucidated.

Overall, although MSC-EVs show promising therapeutic potential for DED, particularly in two published trials, the field currently lacks standardized protocols and publicly available results from most registered studies. Standardization of manufacturing processes, dosing regimens, and outcome measures will be essential for future progress.

### Comparative analysis of MSC-EVs from diverse tissue sources: implications for DED

In the aforementioned experimental and clinical studies, MSC-EVs derived from various sources, such as BMSC-EVs, ADMSC-EVs, UCMSC-EVs, and LGMSC-EVs, were employed. MSCs exhibit phenotype variability depending on their tissue origin, resulting in distinct therapeutic properties, including differences in proliferation rate, differentiation potential, and secretory profiles^[180]^. Accordingly, the protein cargo of MSCs-EVs is strongly influenced by the source of the parent MSCs^[181]^. Therefore, comparing MSC-EVs from different tissue origins becomes necessary to identify the optimal source for treating ocular surface diseases and to facilitate source-directed therapeutic strategies.

Table 3 summarizes the characteristics of MSCs derived from major sources, including their proliferation rates, immunomodulatory effects, osteogenic/adipogenic potential, as well as the cargo features of their derived EVs. The advantages and challenges of these MSC-EVs largely determine their therapeutic potential.

**Table 3:** Comparison of characteristics of MSCs and MSC-EVs from major sources

**Type**	**Proliferation**	**Immunomodulation**	**EV cargo features**	**Special advantages/Notes**	**Ref.**
BM	- Particle yield in 3D comparable to UC (~1E10/mL) - Median particle size 158 nm - Doubling time ~35 h	- BM-EVs enriched in anti-inflammatory proteins (Anxa5, Vcp, S100A4) - Predicted to activate IL-9 signaling, downregulate inflammatory response	- Proteome enriched in Anxa5, Vcp, S100A4 - No source-exclusive proteins - Enriched in bone regeneration-related proteins (POSTN, COL6A2, TTN) - Less enrichment in neuro-regeneration proteins	- Most widely studied, but invasive harvesting, low yield - Donor age affects function - BM-EVs show clear advantages in liver and kidney protection - *In vivo*, preferentially accumulate in BM	[180,182-187]
AD	- Particle yield lower in 3D (~5E9/mL) - Median particle size 143 nm - Doubling time ~34 h	- AD-EVs downregulate inflammation-related pathways (Fn1, Anxa5 downregulation) - In corneal disease models, reduce inflammation	- AD-EVs enriched in Hist1h2ah, Pdia3, Calr, HspA5, Atp5a1, Tpm1, Myh6, Myh9 - Downregulate Fn1, Hspg2, Cxcl12, Col12a1 - Proteome involves oxidative phosphorylation and electron transport chain	- Minimally invasive harvest, high yield - Donor and anatomical site variability affect quality - AD-EVs superior to BM-EVs in pro-angiogenic activity (endothelial sprouting) - Effective in skin wound healing	[182-188]
UC	- Highest particle yield in 3D (~1E10/mL) - Largest median particle size (173 nm) - Doubling time ~22.8 h (WJ) - UCMSCs proliferate rapidly	- UC-EVs exhibit strong immunomodulation, enriched in immune-related proteins - Reduce Th1/Th17 infiltration, promote Tregs - In ocular diseases (dry eye, uveitis), strong anti-inflammatory effects	- UC-EVs enriched in immune regulation proteins - Proteome contains PAI-1 (wound healing, fibrosis) - Rich in Wnt4, promote angiogenesis via β-catenin signaling	- Non-invasive harvest, low immunogenicity, no ethical concerns - Dependent on cord banks, batch variability - *In vivo*, accumulate in spleen, lung, kidney, lymph nodes - High drug loading/delivery capacity - First clinical study in chronic kidney disease	[183-185,187-190]
DP	- Doubling time ~24 h (rapid growth)	- DPSC-EVs promote M2 macrophage polarization, reduce proinflammatory cytokines - Immunomodulatory and antiinflammatory effects	- DPSC-EVs enriched in nervous system-related proteins - High enrichment in neuro-regeneration proteins (e.g., DLGAP1) - High drug loading/delivery capacity similar to UC	- Minimally invasive (teeth) - Neural crest origin, beneficial for neural regeneration - DPSC-EVs show higher uptake by neuronal cells and brain distribution - Promising for maxillofacial and periodontal regeneration	[183-185,191]
WJ	N/A (subset of UC) - MSC doubling time ~22.8 h	- WJMSC-EVs potent immunomodulation, enriched in immune proteins (PZP, FPR2, C3, TLR1)	- Highest protein abundance and most exclusive proteins (12 unique) - Enriched in liver regeneration proteins (ITIH3, N4BP2) - Enriched in cardiac regeneration proteins (PRR14, CASQ2, TUBB1) - Enriched in neuro-regeneration proteins (TMOD2, BRINP2, UNC80)	- Source is UC WJ, non-invasive harvest - High protein expression, suitable for large-scale production - Potential in liver, cardiac, neural, and bone regeneration	[183]
Placenta	Not available	- Placental MSC-EVs have anti-inflammatory and pro-angiogenic effects - Reduce inflammation in liver fibrosis and spinal cord injury models	- High expression of surface markers CD8, CD19, ROR1, CD4, CD209, CD45 - Markers associated with immune/antigenpresenting cells	- Scalable, low immunogenicity source - Combined anti-fibrotic and anti-inflammatory effects - Some ethical concerns	[189-191]

3D: Three-dimensional; AD: adipose; Anxa5: annexin A5; Atp5a1: ATP synthase F1 subunit alpha 1; BM: bone marrow; BRINP2: BMP/retinoic acid inducible neural specific protein 2; C3: complement component 3; Calr: calreticulin; CASQ2: calsequestrin 2; CD4: cluster of differentiation 4; CD8: cluster of differentiation 8; CD19: cluster of differentiation 19; CD45: cluster of differentiation 45; CD209: cluster of differentiation 209; COL6A2: collagen type VI alpha 2 chain; Col12a1: collagen type XII alpha 1 chain; Cxcl12: C-X-C motif chemokine ligand 12; DLGAP1: discs large-associated protein 1; DP: dental pulp; DPSC: dental pulp stem cells; EVs: extracellular vesicles; Fn1: fibronectin 1; FPR2: formyl peptide receptor 2; Hist1h2ah: histone cluster 1 H2A family member H; HspA5: heat shock protein A5; Hspg2: heparan sulfate proteoglycan 2; IL-9: interleukin-9; ITIH3: inter-alpha-trypsin inhibitor heavy chain 3; MSC: mesenchymal stem cell; Myh6: myosin heavy chain 6; Myh9: myosin heavy chain 9; N4BP2: NEDD4 binding protein 2; PAI-1: plasminogen activator inhibitor-1; Pdia3: protein disulfide isomerase A3; POSTN: periostin; PRR14: proline-rich protein 14; PZP: pregnancy zone protein; ROR1: receptor tyrosine kinase-like orphan receptor 1; S100A4: S100 calcium-binding protein A4; Th1: T cell helper type 1; Th17: T cell helper type 17; TLR1: Toll-like receptor 1; TMOD2: tropomodulin 2; Tpm1: tropomyosin 1; Tregs: regulatory T cells; TTN: titin; TUBB1: tubulin beta 1 chain; UC: umbilical cord; UNC80: Unc-80 homolog; Vcp: valosin-containing protein, transitional endoplasmic reticulum ATPase; WJ: Wharton’s jelly; Wnt4: wingless-type MMTV integration site family member 4.

Among the various sources, UCMSC-EVs have attracted particular interest for DED and appear as one of the most frequently used interventions in the currently registered clinical trials. Based on the features listed in Table 3, UCMSC-EVs stand out as a promising candidate for clinical translation for the treatment of DED. They can be harvested non-invasively and exhibit high efficacy, low immunogenicity, and strong proliferative capacity, all favorable features for large-scale production. However, head-to-head comparative studies of MSC-EVs from different sources for DED treatment are still missing, representing a key knowledge gap that warrants future investigation.

### Translational challenges in MSC-EV therapy for DED and corresponding strategies

MSC-EV therapy offers two fundamental advantages for the treatment of DED. (1) Targeted delivery capability: due to their small size, low immunogenicity, high biocompatibility, and the capacity to cross biological barriers and home to sites of injury or inflammation, MSC-EVs constitute an ideal vehicle for targeted topical administration^[5,21,107,192]^; (2) Favorable safety profile: their cell-free nature and inherent lack of self-replication capability substantially minimize the risk of tumorigenicity, thereby conferring a markedly enhanced safety profile compared with cell-based therapies^[100,101]^. Collectively, these intrinsic properties position MSC-EVs as a promising, safe, and effective therapeutic strategy for DED, with strong potential for clinical translation. However, several key translational challenges must be addressed before large-scale clinical application of MSC-EVs can be realized. These challenges primarily stem from the intrinsic complexity of EV biogenesis, the heterogeneity of their molecular cargo, and the unique physiological barriers associated with ocular surface drug delivery. Overcoming these obstacles will be critical for ensuring the safety, reproducibility, and therapeutic reliability of MSC-EVs-based treatments.

#### Ocular-specific anatomical and physiological barriers in translation

The first major translational challenge is overcoming the anatomical and physiological barriers inherent to the eye, particularly the rapid clearance of topically applied drugs by the tear film^[153,193]^. For this reason, drug formulations, delivery devices, and administration routes need to be tailored to the specific therapeutic strategy to overcome these obstacles while ensuring adequate bioavailability. The goal is to increase ocular drug retention, slow down drug elimination, and ultimately improve treatment effectiveness^[193]^.

The tear film plays a critical role in maintaining ocular immune privilege and homeostasis, but it also limits the permeation and extended residence of conventional drug formulations, posing a significant challenge for topical ocular drug delivery. The mucus layer, aqueous layer, and corneal epithelium collectively influence the retention time, adsorption pattern, and penetration route of EVs on the ocular surface^[153,194]^. Once a topical formulation reaches the precorneal pocket - the primary site for absorption and/or pharmacological action - it is rapidly cleared by ocular protective processes such as blinking, basal and reflex tearing, and drainage through the nasolacrimal system, resulting in low bioavailability and uneven distribution^[195,196]^. Despite their small size and high biocompatibility, EVs are not exempt from these challenges.

Some advanced platforms, including hydrogels, nanoparticles, microneedles, and contact lens-based delivery systems, have shown promise in improving ocular retention and controlled release of EVs^[161-163]^.

Biomaterial-assisted EV delivery systems to prolong retention Encapsulating EVs with mucoadhesive materials, such as thermosensitive chitosan or hyaluronic acid hydrogels, can enhance resistance to tear clearance, prolong ocular surface retention, and improve bioavailability^[159,160,181]^. For example, the 3D-Exo-hydrogel system uses a photocurable gelatin methacryloyl (GelMA) hydrogel that rapidly gels upon visible-light irradiation, firmly adheres to the corneal stroma, and effectively fills defects. This system provides mechanical support while creating a stable seal that resists displacement. The porous structure of the hydrogel allows sustained release of 3D-cultured MSC exosomes (3D-Exos) over several days, maintaining therapeutic concentrations at the injury site without the need for repeated administration. Compared to 2D-Exos, 3D-Exos exhibit higher yield, enhanced anti-inflammatory and pro-proliferative effects, and superior capacity for tissue remodeling. By combining durable wound stabilization with prolonged exosome delivery, this cell-free system effectively reduces inflammation, promotes re-epithelialization, restores limbal stem cell function, minimizes scarring, and accelerates overall corneal healing^[197]^.

Surface engineering of EVs for enhanced penetration and retention The negative surface charge of EVs limits their penetration through the similarly negatively charged corneal stroma, which is rich in proteoglycans and collagens. Modifying exosomes with cationic motifs has been shown to significantly improve both corneal retention and penetration following topical application. For example, anchoring arginine-rich cationic peptides (CPC) or avidin via polyethylene glycol (PEG) 2000 lipid insertion neutralizes the native anionic surface charge of milk-derived exosomes from approximately -22 to -2 mV. The resulting weakly cationic surface enables reversible electrostatic binding to anionic glycosaminoglycans in the corneal stroma, reducing washout by the tear film and prolonging corneal retention. In addition, the cationic motifs induce a Donnan partitioning effect (K > 1 for Exo-CPC), increasing exosome accumulation at the corneal interface, while hydrophilic PEG chains facilitate diffusion through the stroma, resulting in a two-fold increase in steady-state diffusivity compared to unmodified exosomes^[198]^. Similarly, incorporation of ε-polylysine-polyethylene glycol-distearoyl phosphatidylethanolamine (PPD) into EV membranes reverses their ζ-potential, enabling enhanced penetration and retention within negatively charged extracellular matrices. This strategy has significantly improved therapeutic efficacy in osteoarthritis and may be directly translatable to corneal applications^[199]^. Overall, reversing EV surface charge toward a cationic state represents a promising approach to enhance both the penetration and retention of topically applied EV-based eye drops in the cornea^[181]^.

Targeting ligands, such as arginine-glycine-aspartic acid (RGD) peptides, can further enhance the selective uptake of EVs and their cargo by corneal cells through integrin-mediated binding, improving targeted delivery to corneal tissues^[181]^. Dual-engineered nanoparticles, coated with MSC membrane vesicles and functionalized with Trans-Activator of Transcription (TAT) cell-penetrating peptides, demonstrate increased corneal permeability, strong adhesion to the ocular surface, and active homing to corneal neovascular endothelial cells. This combined functionality enables high local drug accumulation and sustained therapeutic effects^[200]^.

#### Optimizing MSC-EV administration for ocular surface targeting

The second major challenge is systematically elucidating the pharmacological properties of MSC-EVs, such as bioavailability, targeting ability, pharmacokinetics, and biodistribution. A comprehensive understanding of these properties is essential for designing optimal administration regimens, including the appropriate dosage, delivery route, and treatment frequency to achieve precise and efficient targeting of ocular surface tissues^[5,102,134,201-203]^. EVs can be administered through multiple routes, including intravenous, subconjunctival, subcutaneous, intraperitoneal, oral, intranasal, or intraocular delivery, with the choice depending on the therapeutic goal and the target tissue^[181,203]^. Exosome absorption varies considerably depending on the route of administration, injection site, and dosage, all of which directly influence their therapeutic efficacy^[125,203]^.

Knowledge of the pharmacokinetic behavior of EVs in the chronically inflamed ocular surface microenvironment remains limited, particularly in the context of DED. Inflammatory alterations may significantly affect EV retention time, biodistribution, and biological activity. A robust, context-specific understanding of EV pharmacokinetics is therefore needed for the rational design of dosing regimens and treatment schedules. Addressing this knowledge gap will be critical for advancing EV-based therapies toward clinical translation and warrants greater focus in future studies.

#### Long-term therapeutic profile and safety of MSC-EVs

The third major challenge is thoroughly establishing the long-term therapeutic profile of MSC-EVs. Their efficacy and safety need to be validated through comprehensive clinical trials, with particular emphasis on evaluating potential cumulative effects and unanticipated biological consequences^[134]^.

#### Standardization of Good Manufacturing Practice-compliant production pipelines for MSC-EVs

The fourth major challenge is establishing standardized Good Manufacturing Practice (GMP) production pipelines^[5,104,153,192,203-210]^, including standardization of production processes (isolation, purification, extraction, and scalable production) and standardization of quality control (characterization, identification methods, protocols, and evaluation systems). Several key issues need to be addressed: (1) Control of heterogeneity and standardization of isolation: MSC-EVs exhibit inherent heterogeneity due to variations in their parental MSCs, cell passages, target cells, and the surrounding pathological environment^[5,102,106,125,181,211]^. Consequently, exosomes obtained by different isolation methods vary in yield, purity, protein content, and membrane integrity. Whether these methodological differences impact the immunosuppressive function of MSC-Exos remains unclear^[125]^. Currently, no unified gold standard exists for exosome isolation and purification, and commonly used techniques remain suboptimal with regard to sample purity, processing volume, and time efficiency^[102,209]^. Therefore, standardized protocols for the collection, isolation, purification, and characterization of MSC-EVs should be established to fully elucidate their therapeutic potential, enable precise differentiation of EV subpopulations, and facilitate the development of standardized exosome potency assays. Such standardization is crucial to ensure comparability across studies, as well as to guarantee the quality, safety, and batch-to-batch consistency required for clinical applications; (2) Development of scalable manufacturing processes: Naturally produced exosomes generally exhibit low yields, and their isolation and purification efficiencies are often suboptimal, posing significant challenges for large-scale production^[102,192]^. Moreover, current extraction processes frequently struggle to ensure consistent product purity and yield^[212]^. To enable scalable EV production, it is essential to establish stable, cost-effective, and scalable production and purification processes. These efforts should be supported by advanced analytical techniques such as particle counting, size-based separation methods, and phenotypic characterization to ensure rigorous quality control^[115,213]^.

#### Storage conditions and cryopreservation strategies for MSC-EVs

The fifth major challenge is establishing stable and reproducible storage conditions that preserve the therapeutic potential of EVs^[102,203,211,214,215]^. The effects of different temperature ranges on the stability and bioactivity of EVs require systematic evaluation. Although numerous studies have examined the impact of storage temperature, freeze-thaw cycles, pH, and storage duration, the results remain inconsistent. Nevertheless, most studies suggest that storage at either 4, -20, or -80 °C all meet the requirements for EV preservation, with the optimal conditions depending on the intended application and storage duration. Although it has been reported that EVs remain relatively stable after multiple freeze-thaw cycles, minimizing such cycles is advisable to prevent degradation of bioactive cargo and loss of immunosuppressive effects^[125]^. Storage duration should be determined according to the study design and temperature conditions: 4 °C is preferred for short-term storage, whereas -80 °C is recommended for long-term storage^[102,210]^. A deeper understanding of how storage conditions affect EV structure, function, and biological activity is still needed to refine storage protocols and support the development of effective cryopreservation strategies.

#### Toxicological and microbiological safety assessments for EV sterility

The sixth major challenge is conducting rigorous toxicological and microbiological assessments to ensure the sterility of EV preparations. The potential presence of pathogens - such as bacteria, viruses, or fungi - in these products poses a significant risk of infection^[203,216]^. In response to reported cases of sepsis, the International Society for Extracellular Vesicles has issued safety alert guidelines concerning unproven EV-based therapies^[217]^.

#### Regulatory standardization and safety oversight for therapeutic application of EVs

The final major challenge is establishing a globally unified regulatory framework to standardize the therapeutic application of EVs and ensure mandatory reporting of adverse events^[203]^. In the United States and Europe, EVs are classified as drugs or biological products that require regulatory approval. Despite this, numerous businesses and clinics in these regions continue to promote unapproved exosome-based interventions for indications such as anti-aging, hair loss, autism, rheumatic diseases, and Parkinson’s disease. In contrast, in Japan EVs, including exosomes, are not legally defined as cellular products and therefore fall outside the scope of the Act on the Safety of Regenerative Medicine (ASRM)^[217]^. These inconsistences highlight the urgent need for unified legislative standards, prohibition of unregulated profit-driven human applications to ensure patient safety.

## CONCLUSION AND FUTURE PERSPECTIVES

Continued research to overcome critical translational bottlenecks is expected to accelerate the transition of MSC-EV therapy from experimental research to clinical application. By synergistically leveraging their multifaceted mechanisms, including inflammation regulation, immune modulation, tissue repair and regeneration MSC-EVs have the potential to shift the therapeutic paradigm for DED from symptomatic management toward targeted regenerative therapy, positioning them as a promising and potentially transformative strategy for the management of refractory DED. Nevertheless, most current evidence remains preclinical, and further validation in human-relevant models and clinical studies is required before definitive conclusions regarding their efficacy and safety can be drawn.

## DECLARATIONS

### AI and AI-assisted tools statement

During the preparation of this manuscript, the AI tool ChatGPT (OpenAI, GPT-5.3 Instant model) was used solely to assist in the creation and editing of figures. The tool did not influence the study design, data collection, analysis, interpretation, or the scientific content of the work. All authors take full responsibility for the accuracy, integrity, and final content of the manuscript.

### Copyright

© The Author(s) 2026.
